# Trends in the burden of HPV-associated cancers in Mexico: An analysis from 2011 to 2019

**DOI:** 10.1371/journal.pone.0335307

**Published:** 2025-11-13

**Authors:** Juan Carlos Orengo, Ana Luiza Bierrenbach, Carlos Eduardo Aranda Flores, Elsa Diaz Lopez, Julio Cesar Barbour Oliveira, Rodrigo Gonçalves Queijo, Cintia Irene Parellada

**Affiliations:** 1 MSD (IA) LLC, Guaynabo, Puerto Rico, United States of America; 2 Precision Data, São Paulo, São Paulo, Brazil; 3 Hospital General de Mexico Dr. Eduardo Liceaga, Mexico City, Mexico; 4 The International Federation for Cancer Prevention and Colposcopy (IFCPC), Mexico City, Mexico; 5 MSD Brazil, São Paulo, São Paulo, Brazil; University of Miami, UNITED STATES OF AMERICA

## Abstract

Human papillomavirus (HPV) is a major public health concern, responsible for multiple types of cancer. This study aimed to provide an overview of the burden and temporal trends of HPV-associated cancers in Mexico using national hospital discharge and mortality databases from 2011–2019, including cervical, vulvar, vaginal, penile, anal, oropharyngeal, oral cavity, and laryngeal cancers. Hospitalization and mortality rates per 100,000 population were estimated; HPV-attributable fractions applied, and age-standardized temporal trends evaluated using joinpoint regression. Cervical cancer was the leading contributor, accounting for 88.5% of hospitalizations and 90.9% of HPV-attributable deaths. Hospitalization rates for cervical cancer increased between 2011–2014 (APC = 7.5%, 95% CI: 2.3, 18.0), then declined (APC = −3.0%, 95% CI: −7.8, −0.9). Other HPV-associated cancers had lower hospitalization rates, generally below 1 per 100,000, except for penile and head and neck cancers in males. Significant increases in hospitalization rates were observed in males for anal cancer from 2011–2019 (APC = 7.1%, 95% CI: 0.8, 15.1) and oropharyngeal cancer from 2017–2019 (APC = 18.0%, 95% CI: 4.0, 31.6), while in females, for vaginal cancer from 2017–2019 (APC = 30.7%, 95% CI: 10.6, 45.3) and oral cavity cancer from 2011–2019 (APC = 8.4%, 95% CI: 2.4, 29.1). Mortality for most cancers showed decreasing or stable trends over the study period, except for vulvar cancer in females (AAPC = 1.9%, 95% CI: 0.4, 4.1) and oropharyngeal cancer in both sexes (AAPC = 4.0%, 95% CI: 0.7, 8.0). Across most cancers, males were hospitalized at older ages but died younger than females, except for anal cancer. Overall, the burden of HPV-associated cancers is substantial. While cervical cancer remains prevalent and requires continued elimination efforts, the rising burden of anal and oropharyngeal cancers among males, highlights the need to strengthen public health strategies and raise awareness of HPV’s broader impact across both sexes.

## Introduction

Human papillomavirus (HPV) is a significant global public health concern, associated with conditions such as genital warts and multiple cancer types affecting both sexes [1]. It is estimated that nearly all sexually active individuals will be exposed to HPV at some point in their lives, with the possibility of multiple infections occurring throughout life [2,3]. While most HPV infections are transient and resolve spontaneously, persistent infections with high-risk types can lead to precancers and cancers [1,4]. According to the International Agency for Research on Cancer, HPV is linked to several cancers, such as cervical, vulvar, vaginal, anal, penile, oropharyngeal, oral cavity, and laryngeal cancers [5].

Globally, these HPV-associated cancers are responsible for approximately 620,000 new cancer cases in women and 70,000 in men annually, accounting for almost 5% of cancers [1,6]. In Mexico, the burden of HPV-associated cancers is particularly significant. In 2022, there were an estimated 15,095 new cases and 7,122 deaths in Mexico, with cervical cancer being the most significant contributor [7]. The economic impact is also considerable, encompassing both direct medical costs for treatment and indirect costs such as lost productivity due to illness and premature death [8]. This substantial clinical, societal and economic burden highlights the urgent need for effective interventions to combat these cancers.

To control and eliminate HPV-associated cancers globally, including middle-income countries like Mexico, a comprehensive and coordinated strategy is essential. This includes scaling up HPV vaccination, enhancing screening, improving treatment access, and strengthening health systems [9,10]. In 2020, the World Health Organization outlined specific targets for cervical cancer elimination: achieving 90% HPV vaccination coverage by age 15, 70% twice lifetime coverage screening, and 90% of cases treated by 2030 [11]. If these preventive strategies are implemented, these benefits will extend for other HPV-associated cancers, mainly when males are considered [12,13].

In Mexico, the HPV vaccine was partially introduced in the public National Immunization Program in 2008, focusing on girls aged 12–16 years in municipalities with lower human development indices and high cervical cancer incidence. Since 2012, the vaccine has been administered nationwide primarily to fifth-grade girls, which correspond to ages 11–12. The quadrivalent HPV vaccination program expanded in 2022 to include other priority populations, such as adolescent girls with delayed vaccination, young women aged 9–19 years who have experienced sexual assault as part of a formal care guideline, and cisgender and transgender women and men living with HIV [14]. The 2018 National Health Survey reported that 43.7% of female adolescents aged 10–19 had received at least one HPV vaccine dose, with coverage increasing from 14% at age 11 to 69.1% at age 15 [15].

Gathering comprehensive information about the impact caused by HPV-associated cancers is crucial for guiding effective interventions in Mexico for females and males. This study aimed to quantify the burden and temporal trends of HPV-associated cancers in Mexico from 2011–2019 using nationwide hospitalization and mortality databases. We also estimated HPV-attributable cases and deaths to assess the potential impact of the HPV vaccination program. These findings are intended to inform healthcare policy and clinical practices, supporting decision-makers in enhancing preventive measures to advance efforts to eliminate HPV-associated cancers in Mexico.

## Materials and methods

### Study design and data sources

This retrospective observational study, including descriptive and time-trend analyses, utilized national secondary data on HPV-associated cancers in women and men from January 1, 2011, to December 31, 2019. The national databases used in this study included the Automated System of Hospital Discharges *(Sistema Automatizado de Egresos Hospitalarios*, SAEH), the Sectoral Hospital Discharge database covering the Mexican Institute of Social Security (*Instituto Mexicano del Seguro Social*, IMSS) and the Institute for Social Security and Services for State Workers (*Instituto de Seguridad y Servicios Sociales de los Trabajadores del Estado*, ISSSTE), and the mortality database of the National Institute of Statistics, Geography, and Informatics (*Instituto Nacional de Estadística, Geografía e Informática*, INEGI).

The SAEH primarily includes discharges from Ministry of Health facilities, serving individuals without social security coverage, covering around 36–40% of the Mexican population. IMSS and ISSSTE together cover approximately 55–60% of the population. Combined, SAEH and the sectoral database are estimated to capture approximately 71% of the total population and up to 96% of those with public health coverage [16,17]. Mortality data, sourced from the INEGI, are estimated to capture nearly 100% of all deaths nationwide, covering the same period.

Population data were obtained from the National Population Council (*Consejo Nacional de Población*) using their “Demographic Reconciliation 1950-2019” [18]. All databases are de-identified and publicly available. Additional details on data sources and the Python scripts used to extract hospitalization and mortality data are provided in the Supporting Information (S1 Appendix, S2 Appendix, and S3 Appendix).

### Definitions

Hospital discharge and death data were identified using International Classification of Diseases, 10^th^ Revision (ICD-10) codes for cervical (C53.0-C53.9), vulvar (C51.0-C51.9), vaginal (C52), penile (C60.0-C60.9), and anal cancers (C21.0-C21.8), as well as types of head and neck cancer, which include oral cavity (C02.0-C02.3, C03.0-C03.9, C04.0-C04.9, C05.0, C06.0-C06.9), oropharyngeal (C01, C02.4, C05.1, C05.2, C09.0-C09.9, C10.0, C10.2-C10.9), and laryngeal cancers (C32.0-C32.9) (S1 Table). For hospitalization data, the main ICD-10 diagnosis was used, while for mortality data, the underlying cause of death was considered. The category “all HPV-associated cancers” included all these cancers. Additionally, head and neck cancer types were analyzed both individually and in combination.

HPV-associated cancers were defined as those occurring at anatomic sites where HPV-DNA is frequently detected, regardless of its presence in individual cases. HPV-attributable cancers were defined as those likely caused by HPV. To estimate the attributable fraction for each cancer, we reviewed the most recent literature with robust methodology, prioritizing studies with large sample sizes, national or regional representativeness, and contemporary data. These studies used polymerase chain reaction (PCR) to detect HPV-DNA in tumor samples, and, for selected sites such as vulvar and specific types of head and neck cancers, p16^INK4a^ overexpression was also considered. The HPV-attributable fraction was defined by the proportion of samples positive for these biomarkers. Cervical and anal cancers were considered fully attributed to HPV, with a 100% association [19]. Vulvar cancer had a 48% HPV-attributable fraction in individuals under 60 years of age, and 15% in those aged 60 and older [19]. Vaginal cancer had a fraction of 78%, and penile cancer was estimated at 51% [19]. For types of head and neck cancer, the HPV-attributable fractions were: 9.6% for oral cavity cancer, 39.2% for oropharyngeal cancer, and 14.7% for laryngeal cancer [20] (S2 Table).

### Data analysis

Primary outcomes included the total number of HPV-associated hospitalizations and deaths over the 9-year study period (2011–2019), average annual numbers, corresponding crude rates per 100,000 population, and age-standardized time-trend changes in rates over the same period. Only the first hospitalization event per patient per year was considered to avoid counting multiple hospitalizations for the same patient within a single year. Analyses were stratified by cancer type, age group (categorized as <20, 20–29, 30–39, 40–49, 50–59, 60–69, 70–79, and >80), sex, and year.

As secondary outcomes, HPV-attributable hospitalizations and deaths, average annual numbers, and rates per 100,000 population from 2011–2019 were estimated by applying the attributable fractions to the total number of events. The age distribution and median age at first hospitalization and death were calculated for each type of cancer and sex.

Rates were expressed per 100,000 population. Crude rates were calculated by dividing the number of events by the corresponding annual population estimates. Age- standardized rates were estimated using the World Health Organization standard population to account for shifts in Mexican population age structure over time [21].

Temporal trends in age-standardized hospitalization and mortality rates were analyzed using Joinpoint Regression, a method that identifies statistically significant changes in trends—referred to as joinpoints—allowing the study period to be divided into distinct linear segments with potentially different slopes [22]. Model selection followed the Weighted Bayesian Information Criterion (WBIC), which offers superior computational efficiency, and a data-adaptive alternative approach that balances goodness-of-fit and model simplicity, thereby ensuring the detection of meaningful trend changes [23]. For a 9-year span, the software permits a maximum of one joinpoint. The model selection process evaluates all models from 0 to the maximum number of joinpoints and selects the one with the lowest WBIC value [24,25]. For each segment, the Annual Percentage Change (APC) with corresponding 95% confidence intervals (CIs) was estimated.

The APC measures the rate of change per year within a specific segment, while the Average Annual Percentage Change (AAPC) summarizes the overall trend across the entire period. When no changes (joinpoints) are detected, the APC and AAPC values are identical. Statistical significance was defined as *p* ≤ 0.05. Segments with statistically significant APC or AAPC values (p ≤ 0.05) were interpreted as indicating increasing or decreasing trends. Non-significant values (p > 0.05) were interpreted as stable trends. All rates were log-transformed before modeling, and analyses were stratified by sex and, where possible, by age group.

This study did not require Institutional Review Board or Ethics Committee approval because it utilized publicly available and de-identified data. The databases, which are available online, contain no personal identification information. Statistical analyses were performed using the Databricks environment (Databricks SQL Warehouse, version 2024.40), configured for executing SQL queries. Python (version 3.13.0) was additionally employed for data processing and visualization. Joinpoint regression analyses were conducted using the Joinpoint version 5.4.0 (Statistical Research and Applications Branch, National Cancer Institute) [24].

## Results

### HPV-associated and HPV-attributable hospitalizations

**Fig 1:**
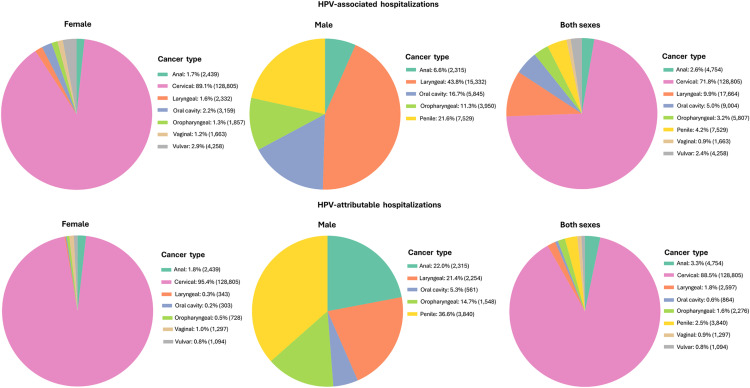
Distribution of HPV-associated and attributable cancer hospitalizations by type and sex, Mexico, 2011-2019.

From 2011–2019, cervical cancer was the primary contributor to HPV-associated hospitalizations among females, accounting for 71.8% of the 179,484 total hospitalizations. The average crude rate for cervical cancer was 22.9 per 100,000 females. Other anogenital HPV-associated cancers had much lower hospitalization rates, ranging from 0.3 per 100,000 for vaginal cancer to 1.4 per 100,000 for penile cancer. Head and neck cancer types showed higher hospitalization rates in males than females: oropharyngeal cancer (0.7 vs. 0.3 per 100,000, 2.3-fold higher), laryngeal cancer (2.8 vs. 0.4 per 100,000, 7-fold higher), and oral cavity cancer (1.1 vs. 0.6 per 100,000, 1.8-fold higher). When combined, head and neck cancer hospitalization rates were 2.9-fold higher in males than females (4.7 vs. 1.3 per 100,000). Anal cancer rates were equal for both sexes at 0.4 per 100,000. When considering all HPV-associated cancers combined, females had a higher overall crude hospitalization rate (25.7 per 100,000) compared to males (6.5 per 100,000), largely driven by the high cervical cancer rates. When applying HPV-attributable fractions, cervical cancer accounted for 88.5% of the 145,527 attributable hospitalizations. Anal cancer contributed 3.3%, penile cancer 2.5%, and head and neck cancers combined 4.0% (Table 1 and Fig 1).

**Table 1:** Hospitalizations for HPV-associated and HPV-attributable cancers by type and sex in Mexico (2011-2019): total numbers, averages, and crude rates per 100,000 population.

Type of cancer	Sex	Total hospitalizations			HPV-attributable hospitalization		
		Total number	Avg annual number	Avg crude rate^a^	Total number	Avg annual number	Avg crude rate^a^
**Cervical cancer**	Female	128,805	14,312	22.9	128,805	14,312	22.9
**Vaginal cancer**	Female	1,663	185	0.3	1,297	144	0.2
**Vulvar cancer**	Female	4,258	473	0.8	1,094	122	0.2
**Penile cancer**	Male	7,529	837	1.4	3,840	427	0.7
**Anal cancer**	Female	2,439	271	0.4	2,439	271	0.4
	Male	2,315	257	0.4	2,315	257	0.4
	Both	4,754	528	0.4	4,754	528	0.4
**Oropharyngeal** **cancer**	Female	1,857	206	0.3	728	81	0.1
	Male	3,950	439	0.7	1,548	172	0.3
	Both	5,807	645	0.5	2,276	253	0.2
**Laryngeal** **cancer**	Female	2,332	259	0.4	343	38	0.1
	Male	15,332	1,704	2.8	2,254	250	0.4
	Both	17,664	1,963	1.6	2,597	288	0.2
**Oral cavity** **cancer**	Female	3,159	351	0.6	303	34	0.1
	Male	5,845	649	1.1	561	62	0.1
	Both	9,004	1,000	0.8	864	96	0.1
**Head and neck** **cancers**	Female	7,348	816	1.3	1,374	153	0.2
	Male	25,127	2,792	4.7	4,363	485	0.8
	Both	32,475	3,608	2.9	5,737	638	0.5
**All HPV-** **associated** **cancers**	Female	144,513	16,057	25.7	135,009	15,002	24.0
	Male	34,971	3,886	6.5	10,518	1,168	1.9
	Both	179,484	19,943	16.3	145,527	16,170	13.2

Avg, average; HPV, human papillomavirus.

^a^Crude rates are expressed per 100,000 population.

### HPV-associated and attributable mortality

**Fig 2:**
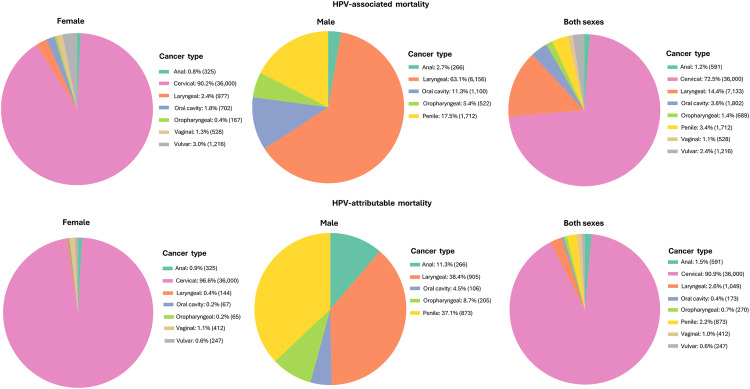
Distribution of HPV-associated and attributable cancer deaths by type and sex, Mexico, 2011-2019.

From 2011 to 2019, cervical cancer caused 36,000 deaths, representing 72.5% of all HPV-associated cancer deaths (49,671 total) and 90.9% of HPV-attributable deaths (26,615 total) across both sexes. The average annual crude mortality rate for HPV-associated cancer was 4.5 per 100,000. Males had 4.2-fold higher mortality rates from head and neck cancers combined than females, with 7,778 male deaths and 1,846 female deaths. HPV-attributable cancer deaths averaged 4,402 deaths annually, with patterns closely mirroring those of HPV-associated cancers, though with lower numbers for cancer types to which a partial attribution fraction was applied (e.g., vulvar, penile, and head and neck cancers) (Table 2 and Fig 2).

**Table 2:** Mortality for HPV-associated and HPV-attributable cancers by type and sex in Mexico (2011-2019): total numbers, averages, and crude rates per 100,000 population.

Type of cancer	Sex	Total deaths			HPV-attributable deaths		
		Total number	Avg annual number	Avg crude rate^a^	Total number	Avg annual number	Avg crude rate^a^
**Cervical cancer**	Female	36,000	4,000	6.4	36,000	4,000	6.4
**Vaginal cancer**	Female	528	59	0.1	412	46	0.1
**Vulvar cancer**	Female	1,216	135	0.2	247	27	0.0
**Penile cancer**	Male	1,712	190	0.3	873	97	0.2
**Anal cancer**	Female	325	36	0.1	325	36	0.1
	Male	266	30	0.1	266	30	0.0
	Both	591	66	0.1	591	66	0.1
**Oropharyngeal** **Cancer**	Female	167	19	0.0	65	7	0.0
	Male	522	58	0.1	205	23	0.0
	Both	689	77	0.1	270	30	0.0
**Laryngeal** **Cancer**	Female	977	109	0.2	144	16	0.0
	Male	6,156	684	1.2	905	101	0.2
	Both	7,133	793	0.6	1,049	117	0.1
**Oral cavity** **Cancer**	Female	702	78	0.1	67	7	0.0
	Male	1,100	122	0.2	106	12	0.0
	Both	1,802	200	0.2	173	19	0.0
**Head and neck** **Cancers**	Female	1,846	206	0.3	276	31	0.0
	Male	7,778	864	1.4	1,216	136	0.2
	Both	9,624	1,070	0.9	1,492	166	0.1
**All HPV-** **associated** **cancers**	Female	39,915	4,436	7.1	37,260	4,139	6.6
	Male	9,756	1,084	1.8	2,355	263	0.4
	Both	49,671	5,520	4.5	39,615	4,402	3.6

Avg, average; HPV, human papillomavirus.

^a^Crude rates are expressed per 100,000 population.

### Age distribution

**Fig 3:**
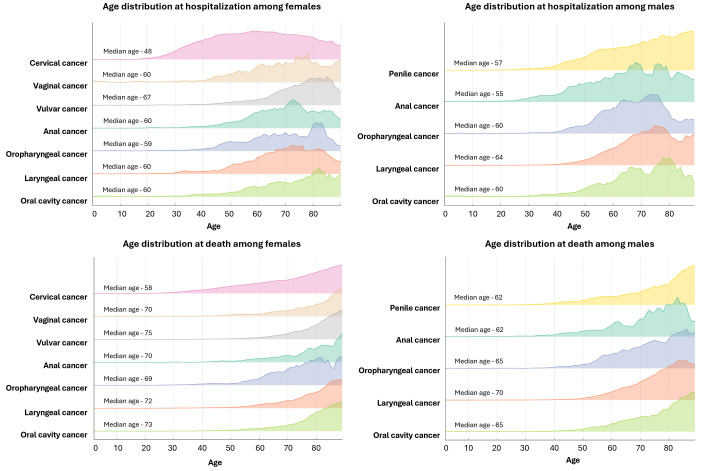
Age distribution and median age at hospitalization and death by HPV-associated cancer type and sex, Mexico, 2011–2019.

In general, males were hospitalized at older ages than females but tended to die at younger ages (Fig 3). However, for anal cancer, males were both hospitalized and died at younger ages compared to females. Among females, cervical cancer hospitalization rates began to rise in the 20–29 age group, peaking in the 50–59 age group, with a median hospitalization age of 48. After this peak, rates declined but remained high among women over 80. Mortality rates rose steadily with age, peaking in the 80 and older age group, with a median age at death of 58.

Other female HPV-associated cancers showed notable hospitalization increases starting in the 40–49 age group, peaking in the 50–59 range. Vulvar cancer hospitalizations rose sharply, with a median age of 67, and continued to increase beyond that age. Vaginal cancer peaked at a median age of 60 before declining. Anal cancer hospitalizations peaked later, around the 70–79 age group, with a median age of 60. For head and neck cancer types in females, the median age ranged between 59 and 60 years. Oral cavity and oropharyngeal cancers in females showed a bimodal distribution, with peaks in the late 50s and again among those aged 80 years and older (Fig 3).

For males, penile cancer hospitalizations began to rise in middle age, peaking in the 70–79 age group, with a median age of 57. Anal cancer hospitalizations peaked earlier than in females, around the 60–69 age group, with a median age of 55. Head and neck cancers in males generally occurred at older ages than in females, with median ages ranging from 60 years for oral cavity and oropharyngeal cancers to 64 years for laryngeal cancer. Mortality patterns in males were generally shifted toward younger ages compared to females, except for anal cancer, where the median age at death (62 years) was similar between sexes.

### Trends on hospitalization and mortality rates for HPV-associated cancers

Among females, the hospitalization rate for cervical cancer showed an upward trend between 2011 and 2014, with an annual increase of 7.5%, followed by a downward trend from 2014 to 2019, with a decrease of 3.0% per year. Overall, the trend was stable across the full period. Vaginal cancer hospitalizations rose sharply between 2017 and 2019, with an annual increase of 30.7%. Vulvar, anal, and oropharyngeal cancers showed no significant changes in hospitalization rates throughout the study period, indicating stable trends. Oral cavity cancer showed a decreasing trend from 2015 to 2019, with an annual reduction of 6.9%, while laryngeal cancer showed a sharper decline of 31.3% per year from 2017 to 2019. For all HPV-associated cancers combined, hospitalization rates increased by 6.8% per year from 2011 to 2014 and decreased by 2.7% per year from 2014 to 2019. Mortality rates among females followed the same pattern of hospitalizations, with significant declines observed for cervical cancer, laryngeal cancer, and all HPV-associated cancers. For other cancer types, mortality trends were stable (Tables 3 and 4).

**Table 3:** Trends in age-standardized hospitalization rates by HPV-associated cancer type and sex (2011–2019): annual percentage change and average annual percentage change.

Type of cancer	Sex	APC % (CI 95%)^a^		AAPC (CI 95%) ^a^
**Cervical cancer**	Female	2011-2014 2014-2019	7.5* (2.6; 18.0) −3.0* (−7.8; −0.9)	0.8 (−0.9; 2.8)
**Vaginal cancer**	Female	2011-2017 2017-2019	−5.5* (−12.7; −2.1) 30.7* (10.6; 45.3)	2.4 (−0.7; 5.0)
**Vulvar cancer**	Female	2011-2016 2016-2019	−4.5 (−15.5; 1.2) 7.3 (−1.8; 21.7)	−0.3 (−3.2; 2.6)
**Penile cancer**	Male	2011-2019	2.0 (−0.2; 4.6)	2.0 (−0.2; 4.6)
**Anal cancer**	Female	2011-2017 2017-2019	−2.6 (−23.8; 24.5) 25.2 (−1.5; 52.3)	3.7 (−2.3; 9.4)
	Male	2011-2019	7.1* (0.8; 15.1)	7.1* (0.8; 15.1)
	Both	2011-2017 2017-2019	1.0 (−10.1; 4.9) 19.8* (6.1; 33.8)	5.4* (2.1; 8.3)
**Oropharyngeal** **cancer**	Female	2011-2019	1.8 (−2.1; 6.1)	1.8 (−2.1; 6.1)
	Male	2011-2017 2017-2019	3.2 (−10.3; 15.3) 18.0* (4.0; 31.6)	6.7* (3.3; 9.9)
	Both	2011-2017 2017-2019	2.6 (−4.0; 4.9) 13.4* (5.5; 21.3)	5.2* (3.1; 6.9)
**Laryngeal** **cancer**	Female	2011-2017 2017-2019	4.6 (−1.1; 42.5) −31.3* (−55.3; −7.4)	−5.8 (−14.3; 4.1)
	Male	2011-2017 2017-2019	2.1 (−0.7; 13.0) −13.6* (−24.2; −2.6)	−2.0 (−4.8; 1.6)
	Both	2011-2017 2017-2019	2.5 (−0.2; 11.5) −15.9* (−26.6; −3.9)	−2.5 (−5.3; 1.1)
**Oral cavity** **cancer**	Female	2011-2015 2015-2019	8.4* (2.4; 29.1) −6.9* (−20.5; −1.7)	0.4 (−3.1; 4.7)
	Male	2011-2019	2.1 (−1.8; 6.6)	2.1 (−1.8; 6.6)
	Both	2011-2019	1.2 (−3.5; 6.5)	1.2 (−3.5; 6.5)
**All HPV-associated cancers**	Female	2011-2014 2014-2019	6.8* (2.3; 16.1) −2.7* (−7.2; −0.8)	0.8 (−0.9; 2.5)
	Male	2011-2019	1.4 (−0.4; 3.4)	1.4 (−0.4; 3.4)
	Both	2011-2014 2014-2019	6.2* (2.1; 14.6) −2.1* (−6.4; −0.3)	1.0 (−0.5; 2.6)

APC, Annual Percentage Change; AAPC, Average Annual Percentage Change; CI, Confidence Intervals.

^a^Trends were classified as increasing or decreasing when APC or AAPC values were statistically significant (p ≤ 0.05), and as stable when non-significant (p > 0.05).

**Table 4:** Trends in age-standardized mortality rates by HPV-associated cancer type and sex (2011–2019): annual percentage change and average annual percentage change.

Type of cancer	Sex	APC % (CI 95%)^a^		AAPC % (CI 95%) ^a^
**Cervical cancer**	Female	2011-2019	−2.3* (−3.2; −1.5)	−2.3* (−3.2; −1.5)
**Vaginal cancer**	Female	2011-2019	2.0 (−4.3; 9.7)	2.0 (−4.3; 9.7)
**Vulvar cancer**	Female	2011-2014 2014-2019	−2.3 (−10.3; 4.8) 4.6 (−1.9; 12.8)	1.9* (0.4; 4.1)
**Penile cancer**	Male	2011-2019	1.2 (−1.8; 4.4)	1.2 (−1.8; 4.4)
**Anal cancer**	Female	2011-2013 2013-2019	−19.2 (−37.6; 15.0) 7.5 (−19.6; 44.0)	0.1 (−5.6; 9.7)
	Male	2011-2019	4.2 (−6.6; 18.6)	4.2 (−6.6; 18.6)
	Both	2011-2019	3.0 (−2.1; 9.1)	3.0 (−2.1; 9.1)
**Oropharyngeal cancer**	Female	2011-2019	7.1 (−4.0; 22.0)	7.1 (−4.0; 22.0)
	Male	2011-2019	2.9 (−1.4; 8.3)	2.9 (−1.4; 8.3)
	Both	2011-2019	4.0* (0.7; 8.0)	4.0* (0.7; 8.0)
**Laryngeal cancer**	Female	2011-2019	−3.9* (−6.0; −1.8)	−3.9* (−6.0; −1.8)
	Male	2011-2019	−5.2* (−7.7; −2.8)	−5.2* (−7.7; −2.8)
	Both	2011-2019	−5.0* (−7.6; −2.5)	−5.0* (−7.6; −2.5)
**Oral cavity cancer**	Female	2011-2014 2014-2019	11.6 (−0.3; 38.5) −1.2 (−16.3; 6.7)	3.4 (−0.7; 8.4)
	Male	2011-2019	0.4 (−5.6; 7.2)	0.4 (−5.6; 7.2)
	Both	2011-2019	1.2 (−4.0; 6.9)	1.2 (−4.0; 6.9)
**All HPV-associated cancers**	Female	2011-2019	−2.0* (−2.6; −1.5)	−2.0* (−2.6; −1.5)
	Male	2011-2019	−2.8* (−4.6; −1.0)	−2.8* (−4.6; −1.0)
	Both	2011-2019	−2.2* (−2.4; −1.9)	−2.2* (−2.4; −1.9)

APC, Annual Percentage Change; AAPC, Average Annual Percentage Change; CI, Confidence Intervals.

^a^Trends were classified as increasing or decreasing when APC or AAPC values were statistically significant (p ≤ 0.05), and as stable when non-significant (p > 0.05).

**Fig 4:**
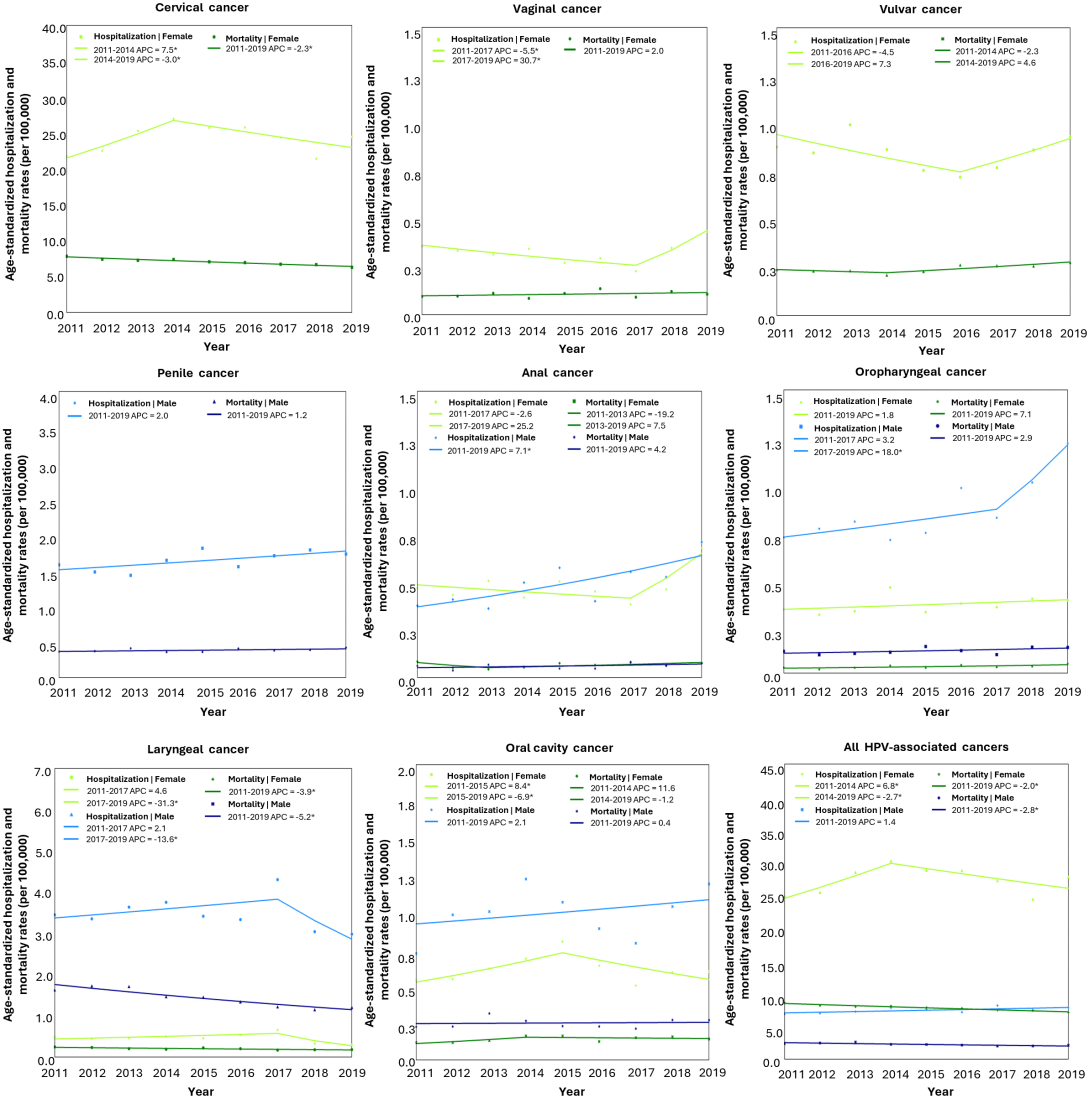
Joinpoint regression trends in age-standardized hospitalization and mortality rates of HPV-associated cancers in Mexico (2011-2019). APC, Annual Percentage Change. Segments with significant APC values are marked with an asterisk; *p* ≤ 0.05.

Among males, hospitalization rates for anal cancer from 2011–2019 and oropharyngeal cancer from 2017–2019 increased annually by 19.8% and 18.0%, respectively. Penile and oral cavity cancers showed stable trends over the period. Laryngeal cancer hospitalizations declined by 13.6% per year from 2017 to 2019. For all HPV-associated cancers combined, hospitalization rates remained stable . Mortality rates declined significantly for laryngeal cancer and all HPV-associated cancers, while trends for other cancer types were stable. Combined-sex analyses revealed significant increases in hospitalization rates for anal and oropharyngeal cancers, and significant declines for laryngeal cancer over the study period. Mortality rates for oropharyngeal cancer increased significantly, while those for laryngeal cancer and all HPV-associated cancers declined (Tables 3 and 4, Fig 4).

Detailed age-standardized rates are provided in S3 and S4 Tables, with complementary crude-rate trends in S5–S7 Tables and S1 Fig. Overall patterns were consistent, but some discrepancies emerged. For cervical cancer, crude rates indicated a significant overall increase (AAPC = 2.4%, 95% CI: 0.6–4.3), as well as for vaginal (AAPC = 4.4%, 95% CI: 0.3–7.5) and penile cancers (AAPC = 4.0%, 95% CI: 1.4–6.9), whereas age-adjusted trends were stable. Regarding mortality, crude rates showed stability for cervical cancer but increases for penile (AAPC = 3.4%, 95% CI: 0.6–6.3) and oropharyngeal cancer in males (AAPC = 5.3%, 95% CI: 0.3–10.8), which were not evident in age-adjusted analyses.

## Discussion

This study provides a comprehensive assessment of HPV-associated and HPV-attributable cancers in Mexico from 2011 to 2019. Cervical cancer remained the leading contributor, accounting for 88.5% of the hospitalizations and 90.9% of HPV-attributable deaths. Age-standardized analysis revealed significant declines in cervical cancer hospitalization and mortality rates over the period, while trends for other HPV-associated cancers varied by site and sex. Males were generally hospitalized at older ages but died younger than females, except for anal cancer. These findings underscore the persistent burden of cervical cancer and the increasing impact of other HPV-associated cancers in males, highlighting the need for expanded prevention and care strategies in both sexes.

Cervical cancer trends likely reflect Mexico’s dedicated secondary prevention programs, which have influenced incidence and mortality. In our study, hospitalizations increased annually by 7.5% from 2011 to 2014, followed by a 3.0% annual decline from 2014–2019. This pattern may result from improvements in diagnostic capacity through Mexico’s National Cervical Cancer Screening Program, which expanded Pap smear coverage in the early 2000s and introduced HPV-DNA testing in 2008. However, access remains uneven, particularly in rural areas with limited social security coverage, which may hinder the full impact of these initiatives [26]. Our findings that the median age for hospitalization was 48 years, and for death, 58 years suggest that many deaths may be linked to diagnoses made before recent improvements. Strengthening diagnostic capacity is essential for timely detection and intervention, which ultimately influence hospitalization and mortality rates [27].

Simultaneously, expanding primary prevention through HPV vaccination is key to accelerating cervical cancer elimination, as recommended by WHO, with potential benefits extending to other HPV-associated cancers [11]. Mathematical models suggest that 90–100% coverage with a broader-valency HPV vaccine, alongside 70% cervical screening coverage, could enable Mexico to reach elimination targets between 2075 and 2080 [12,13]. Multi-age cohort and gender-neutral vaccination strategies, combined with HPV vaccines coveraging the main high-risk HPV types may accelerate progress [12,13], as seen in Australia, where such approaches led to the near-elimination of genital warts and substantial reduction of cervical cancer precursors [28]. In Latin America, including Mexico, HPV types beyond 16 and 18—such as 31, 33, 45, 52, and 58—account for 14.4% to 18.6% of cervical cancer cases [29,30].

Some trend shifts identified by joinpoint analysis likely reflect changes in access to diagnosis and treatment capacity, improvements in referral systems, or evolving risk factor patterns over time. Consistent with these factors, our study found a decline in laryngeal cancer hospitalizations, which may be linked to reduced smoking prevalence, while increases or stabilization in other cancers could result from expanded screening or diagnostic services [31,32]. In Mexico, the centralization of oncology services has been associated with improved survival for some cancers, potentially increasing hospitalization rates while stabilizing or reducing mortality [33]. For example, the observed rise in vaginal cancer hospitalizations in our study may reflect better detection of local recurrence following cervical cancer treatment rather than a true rise in new primary cases. These patterns, along with stable mortality rates, suggest that enhanced surveillance and access to oncology services may be capturing more recurrent disease at the vaginal apex, underscoring the importance of interpreting hospitalization trends within a clinical context [34,35].

Hospitalization and mortality patterns for head and neck cancers in our study varied by site and sex, with mostly stable or decreasing trends except for oropharyngeal cancer in males, which showed an 18% annual increase from 2017 to 2019. These cancers are influenced by multifactorial causes, including lifestyle risks like smoking and alcohol use, both prevalent in Mexican males [36]. While national surveys report declining smoking rates, findings on alcohol use were mixed [37,38]. Globally, HPV-positive oropharyngeal cancers are rising, as seen in the U.S. and parts of Europe [39–42]. Oral HPV prevalence, a relevant risk factor, was found to have similar rates in studies conducted in Mexico, Brazil, and the U.S., though age-specific patterns of HPV prevalence differ, peaking at ages 41–50 years in Brazil (8.3%) and Mexico (6.0%) and at ages 31–40 years in the U.S. (11.0%) [43]. Our findings suggest these trends may reflect a mix of traditional risks and an increasing role of HPV. Public health measures focusing on HPV vaccination, tobacco, and alcohol control are crucial for addressing these trends [13].

Penile cancer showed persistent high hospitalization and mortality rates among males throughout the study period, with no evidence of decline. This sustained burden may reflect low public awareness, delayed healthcare seeking behavior, and sociocultural factors like low circumcision rates and high smoking prevalence [44,45]. Additionally, anal cancer exhibited a significant increase in hospitalization rates among males, who were hospitalized and died at younger median ages than females. These findings partially align with international trends: while anal cancer incidence has increased among females in the U.S. and the Netherlands, rates among males have remained stable overall, contrasting with the increase observed in our study [46,47]. Further research is needed to better characterize the affected male population in our study and to understand how their risk factors may have influenced hospitalization trends. As reported in the literature, anal cancer incidence is notably higher among men who have sex with men, regardless of HIV status [48]. Expanding HPV vaccination and implementing target screening for high-risk groups could help reduce the burden, as demonstrated in other countries [49].

Lastly, our study noticed a pattern in the median age of hospitalization and mortality for HPV-associated cancers, where males were hospitalized at an older age than females but die in a younger age, except for anal cancer in men. A Brazilian study of patients initiating cancer treatment in the public system similarly found that females were younger and 16% less likely to be hospitalized than males [50]. This disparity may be influenced by delayed health-seeking behavior and higher rates of multimorbidity among males. Supporting this, a Brazilian longitudinal aging study reported a stronger association between multimorbidity and hospitalization or readmissions in males compared to females, particularly at older ages [51]. Additionally, a quantitative study in Mexico highlighted gaps in chronic disease management, emphasizing the need for targeted health interventions and preventive measures for men and their families [52].

This study has several strengths that support the robustness of its findings. All data sources used are publicly available and independently verifiable, allowing for reproducibility and external scrutiny. Analytical methods—including joinpoint regression and HPV-attributable fraction estimation—follow established scientific standards and are transparently described in the manuscript. These methodological choices enhance the credibility of our results and ensure that the study can serve as a reliable reference for future research and policy planning.

Nonetheless, some limitations should be acknowledged. First, our analysis combined SAEH with sectoral data, which together cover approximately 71% of the Mexican population and up to 96% of individuals with public health coverage. However, hospitalization rates were calculated using the entire national population, which may attenuate the observed rates and trends, potentially underestimating the true burden in the covered population. This approach may limit the generalizability of our findings, particularly in regions with lower public health coverage [26]. While healthcare coverage remained stable throughout the study period [32,53], gradual improvements in healthcare access, diagnostic capacity, and service quality likely occurred, particularly in preventive care and chronic disease management [54].

Additionally, trend estimates for low-incidence cancers like vaginal and female laryngeal may be unstable due to small sample sizes and annual fluctuations. For instance, female laryngeal cancer showed a 31.3% annual decline from 2017 to 2019 in our analysis, but with a wide confidence interval (−55.3; −7.4), reflecting uncertainty from low counts and a 2017 anomaly. Also, the 9-year study period limits the number of joinpoints that can be modeled, reducing the ability to detect more complex or gradual changes. Lastly, even though we selected large, representative studies from Mexico and Latin America to derive our AFs, these values can vary across settings and over time. As vaccination coverage increases and alters the epidemiology of HPV-associated cancers, updated local estimates will be essential to ensure accurate burden assessments.

The public health implications of our findings are substantial. Sustained efforts in HPV vaccination, screening, and treatment are essential to reduce the burden of HPV-associated diseases. Strengthening these prevention programs will be key to ensuring continued progress in reducing the burden of HPV-related diseases and promoting a comprehensive and equitable approach to cancer prevention in Mexico. Some vulnerable populations, such as men who only have sex with men, may not benefit from herd immunity. Given their elevated risk of HPV-related diseases, excluding men from HPV vaccination raises bioethical concerns. It denies both individual and social protection to a group that is clearly susceptible to infection, disease and viral transmission. Expanding vaccination to include males, as other countries have done, could enhance community protection and help reduce sex disparities in Mexico, especially given the increasing hospitalization and mortality rates among HPV-associated cancers in males. Tailored interventions for both sexes could also facilitate earlier identification and management of HPV-associated conditions, contributing to more effective and inclusive prevention strategies.

## Supporting information

S1 Table International classification of diseases, 10^th^ revision codes for HPV-associated cancers. (DOCX)

S2 Table General methods for the calculation of attributable fraction by cancer site. AF, attributable fraction.^a^Plummer M, de Martel C, Vignat J, Ferlay J, Bray F, Franceschi S. Global burden of cancers attributable to infections in 2012: a synthetic analysis. Lancet Glob Health. 2016;4(9):e609-16. ^b^de Martel C, Georges D, Bray F, Ferlay J, Clifford GM. Global burden of cancer attributable to infections in 2018: a worldwide incidence analysis. Lancet Glob Health. 2020;8(2):e180-e90. ^c^Méndez-Matías G, Velázquez-Velázquez C, Castro-Oropeza R, Mantilla-Morales A, Ocampo-Sandoval D, Burgos-González A, et al. Prevalence of HPV in Mexican Patients with Head and Neck Squamous Carcinoma and Identification of Potential Prognostic Biomarkers. Cancers (Basel). 2021;13(22):5602.
(DOCX)

S3 Table Annual age-standardized hospitalization rates per 100,000 population for HPV-associated cancers by cancer type and sex, Mexico, 2011–2019. (DOCX)

S4 Table Annual age-standardized mortality rates per 100,000 population for HPV-associated cancers by cancer type and sex, Mexico, 2011–2019. (DOCX)

S5 Table Trends in crude hospitalization rates for HPV-associated cancers by cancer type and sex, Mexico, 2011–2019: annual percentage change and average annual percentage change. APC, Annual Percentage Change; AAPC, Average Annual Percentage Change; CI, Confidence Intervals. Segments with significant APC values are marked with an asterisk; *p *≤ 0.05.
(DOCX)

S6 Table Trends in crude mortality rates for HPV-associated cancers by cancer type and sex, Mexico, 2011–2019: annual percentage change and average annual percentage change. APC, Annual Percentage Change; AAPC, Average Annual Percentage Change; CI, Confidence Intervals. Segments with significant APC values are marked with an asterisk; *p *≤ 0.05.
(DOCX)

S7 Table Annual crude hospitalization and mortality rates for HPV-associated cancers by cancer type and sex, Mexico, 2011–2019. (DOCX)

S1 Fig Joinpoint regression trends in crude hospitalization and mortality rates of HPV-associated cancers in Mexico (2011–2019). APC: Annual Percentage Change. Segments with significant APC values are marked with an asterisk; *p *≤ 0.05.
(TIF)

S1 Appendix Data sources. (DOCX)

S2 Appendix Script to extract hospitalization by ICD-10 codes, Mexico, 2011–2019. (DOCX)

S3 Appendix Script to extract mortality by ICD-10 codes, Mexico 2011–2019. (DOCX)
