# Supplementary material for: Trends in the burden of HPV-associated cancers in Mexico: An analysis from 2011 to 2019
Source: PLoS One. 2025 Nov 13;20(11):e0335307. doi: 10.1371/journal.pone.0335307 (PMC12614612; doi:10.1371/journal.pone.0335307)
Supplement: S1 Table — (DOCX) [file pone.0335307.s001.docx]

**S1 Table. International classification of diseases, 10^th^ revision codes for HPV-associated cancers.**

| **ANOGENITAL CANCERS** | |
| --- | --- |
| Anal Cancer | C21 Malignant neoplasm of anus and anal canal |
|  | C21.0 Malignant neoplasm of anus, unspecified |
|  | C21.1 Malignant neoplasm of anal canal |
|  | C21.2 Malignant neoplasm of cloacogenic zone |
|  | C21.8 Malignant neoplasm of overlapping sites of rectum, anus and anal canal |
| Cervical Cancer | C53 Malignant neoplasm of cervix uteri |
|  | C53.0 Malignant neoplasm of endocervix |
|  | C53.1 Malignant neoplasm of exocervix |
|  | C53.8 Malignant neoplasm of overlapping sites of cervix uteri |
|  | C53.9 Malignant neoplasm of cervix uteri, unspecified |
| Penile Cancer | C60 Malignant neoplasm of penis |
|  | C60.0 Malignant neoplasm: Prepuce. |
|  | C60.1 Malignant neoplasm: Glans penis |
|  | C60.2 Malignant neoplasm: Body of penis |
|  | C60.8 Malignant neoplasm: Overlapping lesion of penis |
|  | C60.9 Malignant neoplasm: Penis, unspecified |
| Vaginal Cancer | C52 Malignant neoplasm of vagina |
| Vulvar Cancer | C51 Malignant neoplasm of vulva |
|  | C51.0 Malignant neoplasm of vulva: Labium majus |
|  | C51.1 Malignant neoplasm of vulva: Labium minus |
|  | C51.2 Malignant neoplasm of vulva: Clitoris |
|  | C51.8 Malignant neoplasm: Overlapping lesion of vulva |
|  | C51.9 Malignant neoplasm: Vulva, unspecified |
| **HEAD AND NECK CANCERS** | |
| Laryngeal Cancer | C32 Malignant neoplasm of larynx |
|  | C32.0 Malignant neoplasm of glottis |
|  | C32.1 Malignant neoplasm of supraglottis |
|  | C32.2 Malignant neoplasm of subglottis |
|  | C32.3 Malignant neoplasm of laryngeal cartilage |
|  | C32.8 Malignant neoplasm of overlapping sites of larynx |
|  | C32.9 Malignant neoplasm of larynx, unspecified |
| Oral cavity Cancer | C02.0 Malignant neoplasm of dorsal surface of tongue |
|  | C02.1 Malignant neoplasm of border of tongue |
|  | C02.2 Malignant neoplasm of ventral surface of tongue |
|  | C02.3 Malignant neoplasm of anterior two-thirds of tongue, part unspecified |
|  | C03 Malignant neoplasm of gum |
|  | C03.0 Malignant neoplasm of upper gum |
|  | C03.1 Malignant neoplasm of lower gum |
|  | C03.9 Malignant neoplasm of gum, unspecified |
|  | C04 Malignant neoplasm of floor of mouth |
|  | C04.0 Malignant neoplasm of anterior floor of mouth |
|  | C04.1 Malignant neoplasm of lateral floor of mouth |
|  | C04.8 Malignant neoplasm of overlapping sites of floor of mouth |
|  | C04.9 Malignant neoplasm of floor of mouth, unspecified |
|  | C05.0 Malignant neoplasm of hard palate |
|  | C06 Malignant neoplasm of other and unspecified parts of mouth |
|  | C06.0 Malignant neoplasm of cheek mucosa |
|  | C06.1 Malignant neoplasm of vestibule of mouth |
|  | C06.2 Malignant neoplasm of retromolar area |
|  | C06.8 Malignant neoplasm of overlapping sites of other and unspecified parts of mouth |
|  | C06.9 Malignant neoplasm of mouth, unspecified |
| Oropharyngeal Cancer | C01 Malignant neoplasm of base of tongue |
|  | C02.4 Malignant neoplasm of lingual tonsil |
|  | C05.1 Malignant neoplasm of soft palate |
|  | C05.2 Malignant neoplasm of uvula |
|  | C09 Malignant neoplasm of tonsil |
|  | C09.0 Malignant neoplasm of tonsillar fossa |
|  | C09.1 Malignant neoplasm of tonsillar pillar (anterior) (posterior) |
|  | C09.8 Malignant neoplasm of overlapping sites of tonsil |
|  | C09.9 Malignant neoplasm of tonsil, unspecified |
|  | C10.0 Malignant neoplasm of vallecula |
|  | C10.2 Malignant neoplasm of lateral wall of oropharynx |
|  | C10.3 Malignant neoplasm of posterior wall of oropharynx |
|  | C10.4 Malignant neoplasm of branchial cleft |
|  | C10.8 Malignant neoplasm of overlapping sites of oropharynx |
|  | C10.9 Malignant neoplasm of oropharynx, unspecified |
