# Supplementary material for: Trends in the burden of HPV-associated cancers in Mexico: An analysis from 2011 to 2019
Source: PLoS One. 2025 Nov 13;20(11):e0335307. doi: 10.1371/journal.pone.0335307 (PMC12614612; doi:10.1371/journal.pone.0335307)
Supplement: S2 Table — AF, attributable fraction.aPlummer M, de Martel C, Vignat J, Ferlay J, Bray F, Franceschi S. Global burden of cancers attributable to infections in 2012: a synthetic analysis. Lancet Glob Health. 2016;4(9):e609-16. bde Martel C, Georges D, Bray F, Ferlay J, Clifford GM. Global burden of cancer attributable to infections in 2018: a worldwide incidence analysis. Lancet Glob Health. 2020;8(2):e180-e90. cMéndez-Matías G, Velázquez-Velázquez C, Castro-Oropeza R, Mantilla-Morales A, Ocampo-Sandoval D, Burgos-González A, et al. Prevalence of HPV in Mexican Patients with Head and Neck Squamous Carcinoma and Identification of Potential Prognostic Biomarkers. Cancers (Basel). 2021;13(22):5602. (DOCX) [file pone.0335307.s002.docx]

**S2 Table. General methods for the calculation of attributable fraction by cancer site.**

| Cancer by Anatomic site | Detection Method for prevalence in cases | Population | AF(%) | Reference |
| --- | --- | --- | --- | --- |
| Cervix | PCR | World | 100.0% | Plummer 2016 ^a^ |
| Vagina | PCR | World | 78.0% | Plummer 2016 ^a^ |
| Vulva | PCR + p16INK4A | World | 48.0% < 60 years  15% ≥ 60 years | Plummer 2016 ^a^ |
| Anus | PCR | World | 100.0% | de Martel C 2020^b^ |
| Oral cavity | PCR + p16INK4A | Mexico | 9.6% | Mendez-Matías 2021^c^ |
| Oropharynx | PCR + p16INK4A | Mexico | 39.2% | Mendez-Matías 2021^c^ |
| Larynx | PCR + p16INK4A | Mexico | 14.7% | Mendez-Matías 2021^c^ |
| Penile | PCR | World | 51% | Plummer 2016 ^a^ |

AF, attributable fraction.

^a^Plummer M, de Martel C, Vignat J, Ferlay J, Bray F, Franceschi S. Global burden of cancers attributable to infections in 2012: a synthetic analysis. Lancet Glob Health. 2016;4(9):e609-16.

^b^de Martel C, Georges D, Bray F, Ferlay J, Clifford GM. Global burden of cancer attributable to infections in 2018: a worldwide incidence analysis. Lancet Glob Health. 2020;8(2):e180-e90.

^c^Méndez-Matías G, Velázquez-Velázquez C, Castro-Oropeza R, Mantilla-Morales A, Ocampo-Sandoval D, Burgos-González A, et al. Prevalence of HPV in Mexican Patients with Head and Neck Squamous Carcinoma and Identification of Potential Prognostic Biomarkers. Cancers (Basel). 2021;13(22):5602.
