# Supplementary material for: Trends in the burden of HPV-associated cancers in Mexico: An analysis from 2011 to 2019
Source: PLoS One. 2025 Nov 13;20(11):e0335307. doi: 10.1371/journal.pone.0335307 (PMC12614612; doi:10.1371/journal.pone.0335307)
Supplement: S3 Table — (DOCX) [file pone.0335307.s003.docx]

**S3 Table. Annual age-standardized hospitalization rates per 100,000 population by HPV-associated cancer type and sex, Mexico, 2011–2019.**

| **Type of cancer** | **Sex** | **Year** | | | | | | | | | |
| --- | --- | --- | --- | --- | --- | --- | --- | --- | --- | --- | --- |
|  |  | **2011** | **2012** | **2013** | **2014** | **2015** | **2016** | **2017** | **2018** | **2019** |  |
| **Cervical cancer** | Female | 21.79 | 22.53 | 25.30 | 26.99 | 25.74 | 25.79 | 24.39 | 21.41 | 24.46 |  |
| **Vaginal cancer** | Female | 0.35 | 0.33 | 0.31 | 0.34 | 0.27 | 0.29 | 0.23 | 0.35 | 0.43 |  |
| **Vulvar cancer** | Female | 0.88 | 0.85 | 1.00 | 0.87 | 0.76 | 0.72 | 0.77 | 0.86 | 0.93 |  |
| **Penile cancer** | Male | 1.58 | 1.47 | 1.43 | 1.64 | 1.81 | 1.55 | 1.70 | 1.78 | 1.73 |  |
| **Anal cancer** | Female | 0.47 | 0.43 | 0.51 | 0.42 | 0.50 | 0.45 | 0.38 | 0.46 | 0.68 |  |
|  | Male | 0.38 | 0.41 | 0.36 | 0.50 | 0.58 | 0.40 | 0.55 | 0.53 | 0.71 |  |
|  | Both | 0.43 | 0.42 | 0.44 | 0.46 | 0.54 | 0.43 | 0.46 | 0.49 | 0.69 |  |
| **Oropharyngeal cancer** | Female | 0.34 | 0.31 | 0.33 | 0.45 | 0.32 | 0.37 | 0.35 | 0.39 | 0.39 |  |
|  | Male | 0.72 | 0.77 | 0.80 | 0.71 | 0.74 | 0.98 | 0.82 | 1.01 | 1.22 |  |
|  | Both | 0.52 | 0.52 | 0.55 | 0.57 | 0.52 | 0.65 | 0.57 | 0.68 | 0.78 |  |
| **Laryngeal cancer** | Female | 0.49 | 0.44 | 0.45 | 0.49 | 0.45 | 0.53 | 0.65 | 0.33 | 0.30 |  |
|  | Male | 3.45 | 3.35 | 3.64 | 3.75 | 3.42 | 3.33 | 4.31 | 3.04 | 2.98 |  |
|  | Both | 1.88 | 1.81 | 1.95 | 2.02 | 1.84 | 1.85 | 2.37 | 1.60 | 1.56 |  |
| **Oral cavity cancer** | Female | 0.54 | 0.55 | 0.62 | 0.69 | 0.80 | 0.64 | 0.50 | 0.59 | 0.60 |  |
|  | Male | 0.72 | 0.98 | 1.01 | 1.23 | 1.07 | 0.89 | 0.79 | 1.04 | 1.19 |  |
|  | Both | 0.63 | 0.75 | 0.80 | 0.94 | 0.93 | 0.76 | 0.64 | 0.80 | 0.88 |  |
| **All HPV-associated cancers** | Female | 24.85 | 25.45 | 28.52 | 30.25 | 28.84 | 28.79 | 27.27 | 24.39 | 27.79 |  |
|  | Male | 6.84 | 6.98 | 7.23 | 7.82 | 7.61 | 7.15 | 8.17 | 7.40 | 7.83 |  |
|  | Both | 16.30 | 16.68 | 18.40 | 19.59 | 18.78 | 18.53 | 18.23 | 16.34 | 18.33 |  |
