# Supplementary material for: Trends in the burden of HPV-associated cancers in Mexico: An analysis from 2011 to 2019
Source: PLoS One. 2025 Nov 13;20(11):e0335307. doi: 10.1371/journal.pone.0335307 (PMC12614612; doi:10.1371/journal.pone.0335307)
Supplement: S4 Table — (DOCX) [file pone.0335307.s004.docx]

**S4 Table. Annual age-standardized mortality rates per 100,000 population by HPV-associated cancer type and sex, Mexico, 2011–2019.**

| **Type of cancer** | **Sex** | **Year** | | | | | | | | | |
| --- | --- | --- | --- | --- | --- | --- | --- | --- | --- | --- | --- |
|  |  | **2011** | **2012** | **2013** | **2014** | **2015** | **2016** | **2017** | **2018** | **2019** |  |
| **Cervical cancer** | Female | 7.82 | 7.40 | 7.24 | 7.37 | 7.04 | 6.92 | 6.70 | 6.65 | 6.27 |  |
| **Vaginal cancer** | Female | 0.09 | 0.10 | 0.11 | 0.08 | 0.11 | 0.13 | 0.09 | 0.12 | 0.11 |  |
| **Vulvar cancer** | Female | 0.24 | 0.23 | 0.23 | 0.21 | 0.23 | 0.26 | 0.26 | 0.26 | 0.28 |  |
| **Penile cancer** | Male | 0.36 | 0.37 | 0.41 | 0.35 | 0.35 | 0.40 | 0.38 | 0.38 | 0.42 |  |
| **Anal cancer** | Female | 0.08 | 0.05 | 0.04 | 0.05 | 0.07 | 0.06 | 0.08 | 0.06 | 0.07 |  |
|  | Male | 0.06 | 0.04 | 0.07 | 0.06 | 0.05 | 0.05 | 0.08 | 0.06 | 0.07 |  |
|  | Both | 0.07 | 0.04 | 0.05 | 0.05 | 0.06 | 0.06 | 0.08 | 0.06 | 0.07 |  |
| **Oropharyngeal cancer** | Female | 0.03 | 0.02 | 0.03 | 0.04 | 0.03 | 0.04 | 0.03 | 0.03 | 0.05 |  |
|  | Male | 0.11 | 0.10 | 0.10 | 0.11 | 0.14 | 0.12 | 0.10 | 0.14 | 0.14 |  |
|  | Both | 0.07 | 0.05 | 0.06 | 0.07 | 0.08 | 0.08 | 0.06 | 0.08 | 0.09 |  |
| **Laryngeal cancer** | Female | 0.24 | 0.23 | 0.20 | 0.18 | 0.22 | 0.20 | 0.16 | 0.17 | 0.18 |  |
|  | Male | 1.63 | 1.72 | 1.71 | 1.46 | 1.45 | 1.34 | 1.22 | 1.15 | 1.20 |  |
|  | Both | 0.89 | 0.93 | 0.91 | 0.78 | 0.80 | 0.73 | 0.66 | 0.63 | 0.66 |  |
| **Oral cavity cancer** | Female | 0.12 | 0.12 | 0.13 | 0.16 | 0.16 | 0.12 | 0.15 | 0.16 | 0.14 |  |
|  | Male | 0.22 | 0.23 | 0.31 | 0.26 | 0.23 | 0.23 | 0.21 | 0.27 | 0.27 |  |
|  | Both | 0.17 | 0.17 | 0.22 | 0.21 | 0.19 | 0.17 | 0.18 | 0.21 | 0.20 |  |
| **All HPV-associated cancers** | Female | 8.62 | 8.15 | 7.99 | 8.10 | 7.86 | 7.75 | 7.47 | 7.45 | 7.10 |  |
|  | Male | 2.39 | 2.45 | 2.60 | 2.25 | 2.22 | 2.13 | 1.98 | 2.00 | 2.10 |  |
|  | Both | 5.68 | 5.45 | 5.44 | 5.34 | 5.20 | 5.10 | 4.88 | 4.88 | 4.74 |  |
