# Supplementary material for: Trends in the burden of HPV-associated cancers in Mexico: An analysis from 2011 to 2019
Source: PLoS One. 2025 Nov 13;20(11):e0335307. doi: 10.1371/journal.pone.0335307 (PMC12614612; doi:10.1371/journal.pone.0335307)
Supplement: S5 Table — APC, Annual Percentage Change; AAPC, Average Annual Percentage Change; CI, Confidence Intervals. Segments with significant APC values are marked with an asterisk; p ≤ 0.05. (DOCX) [file pone.0335307.s005.docx]

**S5 Table. Trends in crude hospitalization rates by HPV-associated cancer and sex, Mexico, 2011–2019: annual percentage change and average annual percentage change.**

| **Type of cancer** | **Sex** | **Crude hospitalization rate trends** | | |
| --- | --- | --- | --- | --- |
|  |  |  |  |  |
|  |  | **APC^a^** | | **AAPC^a^**  **(CI 95%)** |
|  |  | **Period** | **% (CI 95%)** | **2011-2019** |
| **Cervical cancer** | Female | 2011-2014  2014-2019 | 9.5* (4.3; 19.8)  -1.7 (-6.3; 0.4) | 2.4* (0.6; 4.3) |
| **Vaginal cancer** | Female | 2011-2017  2017-2019 | -3.8 (-15.6; 0.7)  33.4* (9.1; 51.3) | 4.4* (0.3; 7.5) |
| **Vulvar cancer** | Female | 2011-2016  2016-2019 | -2.5 (-13.5; 2.5)  10.0* (1.2; 24.3) | 2.0 (-0.9; 4.7) |
| **Penile cancer** | Male | 2011-2019 | 4.0* (1.4; 6.9) | 4.0* (1.4; 6.9) |
| **Anal cancer** | Female | 2011-2017  2017-2019 | -0.6 (-22.9; 28.2)  27.9 (-0.0; 56.2) | 5.8 (-0.5; 11.9) |
|  | Male | 2011-2019 | 8.8* (2.4; 17.1) | 8.8* (2.4; 17.1) |
|  | Both | 2011-2017  2017-2019 | 2.9 (-8.2; 6.8)  22.0 (8.2; 36.1) | 7.4* (4.1; 10.2) |
| **Oropharyngeal**  **cancer** | Female | 2011-2019 | 3.7* (0.1; 7.7) | 3.7* (0.1; 7.7) |
|  | Male | 2011-2017  2017-2019 | 5.0 (-8.3; 12.9)  20.7* (6.4; 34.0) | 8.7* (5.3; 11.7) |
|  | Both | 2011-2017  2017-2019 | 4.5 (-1.8; 6.7)  15.6 (7.7; 23.3) | 7.2* (5.1; 8.9) |
| **Laryngeal**  **cancer** | Female | 2011-2017  2017-2019 | 6.9 (-0.7; 61.9)  -30.2* (-57.3; -3.0) | -4.0 (-13.7; 8.6) |
|  | Male | 2011-2017  2017-2019 | 4.5* (1.6; 14.8)  -11.5* (-21.8; -0.2) | 0.2 (-2.4; 3.8) |
|  | Both | 2011-2017  2017-2019 | 4.8* (2.0; 13.6)  -14.0* (-23.6; -1.8) | -0.3 (-2.8; 3.2) |
| **Oral cavity**  **cancer** | Female | 2011-2015  2015-2019 | 9.5* (3.9; 28.8)  -4.8 (-17.7; 0.3) | 2.1 (-1.2; 6.2) |
|  | Male | 2011-2019 | 3.8 (-0.3; 8.4) | 3.8 (-0.3; 8.4) |
|  | Both | 2011-2019 | 2.9 (-1.8; 8.3) | 2.9 (-1.8; 8.2) |
| **All HPV-associated cancers** | Female | 2011-2014  2014-2019 | 8.8* (4.1; 18.2)  -1.3 (-5.6; 0.7) | 2.4* (0.7; 4.1) |
|  | Male | 2011-2019 | 3.5* (1.9; 5.3) | 3.5* (1.9; 5.3) |
|  | Both | 2011-2014  2014-2019 | 8.2* (3.9; 16.7)  -0.6 (-4.9; 1.2) | 2.6* (1.1; 4.2) |

APC, Annual Percentage Change; AAPC, Average Annual Percentage Change; CI, Confidence Intervals. **^a^**Trends were classified as increasing or decreasing when APC or AAPC values were statistically significant (p ≤ 0.05), and as stable when non-significant (p > 0.05).
