# Supplementary material for: Trends in the burden of HPV-associated cancers in Mexico: An analysis from 2011 to 2019
Source: PLoS One. 2025 Nov 13;20(11):e0335307. doi: 10.1371/journal.pone.0335307 (PMC12614612; doi:10.1371/journal.pone.0335307)
Supplement: S6 Table — APC, Annual Percentage Change; AAPC, Average Annual Percentage Change; CI, Confidence Intervals. Segments with significant APC values are marked with an asterisk; p ≤ 0.05. (DOCX) [file pone.0335307.s006.docx]

**S6 Table. Trends in crude mortality rates by HPV-associated cancer type and sex, Mexico, 2011–2019: annual percentage change and average annual percentage change.**

| **Type of cancer** | **Sex** | **Crude mortality rate trends** | | |
| --- | --- | --- | --- | --- |
|  |  |  |  |  |
|  |  | **APC^a^** | | **AAPC^a^**  **(CI 95%)** |
|  |  | **Period** | **% (CI 95%)** | **2011-2019** |
| **Cervical cancer** | Female | 2011-2019 | -0.4 (-1.3; 0.5) | -0.4 (-1.3; 0.5) |
| **Vaginal cancer** | Female | 2011-2019 | 4.6 (-1.8; 12.3) | 4.6 (-1.8; 12.3) |
| **Vulvar cancer** | Female | 2011-2014  2014-2019 | -0.4 (-8.3; 5.8)  7.2* (3.4; 15.3) | 4.3* (2.8; 6.4) |
| **Penile cancer** | Male | 2011-2019 | 3.4* (0.6; 6.3) | 3.4* (0.6; 6.3) |
| **Anal cancer** | Female | 2011-2013  2013-2019 | -18.1 (-37.3; 17.3)  10.1 (-18.4; 48.2) | 2.2 (-3.8; 12.2) |
|  | Male | 2011-2019 | 6.2 (-5.4; 21.8) | 6.2 (-5.4; 21.8) |
|  | Both | 2011-2019 | 5.1* (0.3; 10.8) | 5.1* (0.3; 10.8) |
| **Oropharyngeal**  **cancer** | Female | 2011-2019 | 9.6 (-1.4; 24.6) | 9.6 (-1.4; 24.6) |
|  | Male | 2011-2019 | 5.3* (1.2; 10.2) | 5.3* (1.2; 10.2) |
|  | Both | 2011-2019 | 6.3* (2.7; 10.6) | 6.3* (2.7; 10.6) |
| **Laryngeal**  **cancer** | Female | 2011-2019 | -1.7 (-3.7; 0.4) | -1.7 (-3.7; 0.4) |
|  | Male | 2011-2019 | -2.9* (-5.1; -0.8) | -2.9* (-5.1; -0.8) |
|  | Both | 2011-2019 | -2.7* (-5.1; -0.4) | -2.7* (-5.1; -0.4) |
| **Oral cavity**  **cancer** | Female | 2011-2014  2014-2019 | 13.8* (3.5; 38.0)  1.0 (-12.2; 7.0) | 5.6* (1.9; 10.1) |
|  | Male | 2011-2019 | 2.6 (-4.0; 10.2) | 2.6 (-4.0; 10.2) |
|  | Both | 2011-2019 | 3.4 (-1.4; 8.8) | 3.4 (-1.4; 8.8) |
| **All HPV-associated cancers** | Female | 2011-2019 | -0.0 (-0.6;0.6) | 0.0 (-0.6; 0.6) |
|  | Male | 2011-2019 | -0.5 (-2.1;1.0) | -0.5 (-2.1; 1.0) |
|  | Both | 2011-2019 | -0.2 (-0.4; 0.1) | -0.1 (-0.4; 0.1) |

APC, Annual Percentage Change; AAPC, Average Annual Percentage Change; CI, Confidence Intervals. ^a^Trends were classified as increasing or decreasing when APC or AAPC values were statistically significant (p ≤ 0.05), and as stable when non-significant (p > 0.05). Segments with significant APC values are marked with an asterisk; *p* ≤ 0.05.
