# Supplementary material for: Trends in the burden of HPV-associated cancers in Mexico: An analysis from 2011 to 2019
Source: PLoS One. 2025 Nov 13;20(11):e0335307. doi: 10.1371/journal.pone.0335307 (PMC12614612; doi:10.1371/journal.pone.0335307)
Supplement: S7 Table — (DOCX) [file pone.0335307.s007.docx]

**S7 Table. Annual crude hospitalization and mortality rates by HPV-associated cancer type and sex, Mexico, 2011–2019.**

| **Type of cancer** | **Sex** | **Annual crude rates (per 100,000)** | | | | | | | | |  |
| --- | --- | --- | --- | --- | --- | --- | --- | --- | --- | --- | --- |
|  |  | **Year** | | | | | | | | |  |
|  |  | **2011** | **2012** | **2013** | **2014** | **2015** | **2016** | **2017** | **2018** | **2019** |  |
| **Hospitalization** | | | | | | | | | | |  |
| **Cervical cancer** | Female | 19.25 | 20.31 | 23.24 | 25.21 | 24.30 | 24.77 | 23.67 | 21.11 | 24.45 |  |
| **Vaginal cancer** | Female | 0.30 | 0.28 | 0.28 | 0.31 | 0.24 | 0.27 | 0.22 | 0.33 | 0.43 |  |
| **Vulvar cancer** | Female | 0.72 | 0.71 | 0.85 | 0.75 | 0.68 | 0.65 | 0.72 | 0.83 | 0.91 |  |
| **Penile cancer** | Male | 1.25 | 1.18 | 1.17 | 1.36 | 1.55 | 1.34 | 1.51 | 1.61 | 1.59 |  |
| **Anal cancer** | Female | 0.39 | 0.37 | 0.45 | 0.37 | 0.45 | 0.41 | 0.36 | 0.44 | 0.66 |  |
|  | Male | 0.31 | 0.33 | 0.30 | 0.43 | 0.50 | 0.36 | 0.49 | 0.48 | 0.66 |  |
|  | Both | 0.35 | 0.35 | 0.38 | 0.40 | 0.47 | 0.39 | 0.42 | 0.46 | 0.66 |  |
| **Oropharyngeal cancer** | Female | 0.28 | 0.26 | 0.29 | 0.41 | 0.30 | 0.35 | 0.33 | 0.38 | 0.38 |  |
|  | Male | 0.56 | 0.61 | 0.65 | 0.58 | 0.62 | 0.82 | 0.72 | 0.90 | 1.10 |  |
|  | Both | 0.42 | 0.43 | 0.47 | 0.49 | 0.46 | 0.58 | 0.52 | 0.63 | 0.73 |  |
| **Laryngeal cancer** | Female | 0.41 | 0.37 | 0.39 | 0.43 | 0.41 | 0.49 | 0.63 | 0.31 | 0.29 |  |
|  | Male | 2.59 | 2.58 | 2.83 | 2.99 | 2.78 | 2.79 | 3.71 | 2.67 | 2.68 |  |
|  | Both | 1.48 | 1.45 | 1.59 | 1.68 | 1.57 | 1.62 | 2.14 | 1.47 | 1.46 |  |
| **Oral cavity cancer** | Female | 0.46 | 0.48 | 0.55 | 0.62 | 0.72 | 0.58 | 0.48 | 0.57 | 0.59 |  |
|  | Male | 0.58 | 0.79 | 0.83 | 1.01 | 0.90 | 0.76 | 0.70 | 0.94 | 1.09 |  |
|  | Both | 0.52 | 0.63 | 0.69 | 0.81 | 0.81 | 0.67 | 0.59 | 0.75 | 0.83 |  |
| **All HPV-associated cancers** | Female | 21.82 | 22.79 | 26.04 | 28.10 | 27.09 | 27.53 | 26.41 | 23.98 | 27.71 |  |
|  | Male | 5.30 | 5.50 | 5.79 | 6.37 | 6.35 | 6.07 | 7.12 | 6.59 | 7.11 |  |
|  | Both | 13.74 | 14.33 | 16.13 | 17.47 | 16.94 | 17.02 | 16.96 | 15.46 | 17.62 |  |
| **Mortality** | | | | | | | | | | | |
| **Cervical cancer** | | Female | 6.60 | 6.37 | 6.35 | 6.59 | 6.42 | 6.44 | 6.36 | 6.44 | 6.20 |
| **Vaginal cancer** | | Female | 0.08 | 0.08 | 0.09 | 0.07 | 0.10 | 0.12 | 0.08 | 0.12 | 0.10 |
| **Vulvar cancer** | | Female | 0.19 | 0.19 | 0.19 | 0.18 | 0.20 | 0.24 | 0.24 | 0.24 | 0.27 |
| **Penile cancer** | | Male | 0.28 | 0.29 | 0.32 | 0.29 | 0.30 | 0.34 | 0.33 | 0.34 | 0.38 |
| **Anal cancer** | | Female | 0.07 | 0.04 | 0.04 | 0.05 | 0.07 | 0.06 | 0.07 | 0.06 | 0.07 |
|  |  | Male | 0.05 | 0.03 | 0.05 | 0.05 | 0.04 | 0.04 | 0.07 | 0.05 | 0.07 |
|  |  | Both | 0.06 | 0.03 | 0.04 | 0.05 | 0.05 | 0.05 | 0.07 | 0.06 | 0.07 |
| **Oropharyngeal cancer** | | Female | 0.07 | 0.04 | 0.04 | 0.05 | 0.07 | 0.06 | 0.07 | 0.06 | 0.07 |
|  |  | Male | 0.09 | 0.07 | 0.08 | 0.09 | 0.12 | 0.10 | 0.08 | 0.12 | 0.12 |
|  |  | Both | 0.05 | 0.04 | 0.05 | 0.06 | 0.07 | 0.07 | 0.06 | 0.08 | 0.08 |
| **Laryngeal cancer** | | Female | 0.20 | 0.19 | 0.17 | 0.16 | 0.19 | 0.18 | 0.15 | 0.16 | 0.17 |
|  |  | Male | 1.20 | 1.29 | 1.32 | 1.15 | 1.17 | 1.10 | 1.03 | 1.00 | 1.07 |
|  |  | Both | 0.69 | 0.73 | 0.73 | 0.64 | 0.67 | 0.63 | 0.58 | 0.57 | 0.61 |
| **Oral cavity cancer** | | Female | 0.09 | 0.10 | 0.11 | 0.14 | 0.14 | 0.11 | 0.14 | 0.15 | 0.14 |
|  |  | Male | 0.17 | 0.17 | 0.24 | 0.21 | 0.19 | 0.19 | 0.18 | 0.24 | 0.24 |
|  |  | Both | 0.13 | 0.14 | 0.18 | 0.18 | 0.16 | 0.15 | 0.16 | 0.19 | 0.19 |
| **All HPV-associated cancers** | | Female | 7.25 | 6.98 | 6.98 | 7.22 | 7.14 | 7.19 | 7.08 | 7.20 | 7.00 |
|  |  | Male | 1.78 | 1.86 | 2.02 | 1.78 | 1.80 | 1.77 | 1.69 | 1.75 | 1.88 |
|  |  | Both | 4.57 | 4.47 | 4.55 | 4.56 | 4.53 | 4.53 | 4.44 | 4.53 | 4.49 |
