# Supplementary figures and images for: Trends in the burden of HPV-associated cancers in Mexico: An analysis from 2011 to 2019
Source: PLoS One. 2025 Nov 13;20(11):e0335307. doi: 10.1371/journal.pone.0335307 (PMC12614612; doi:10.1371/journal.pone.0335307)

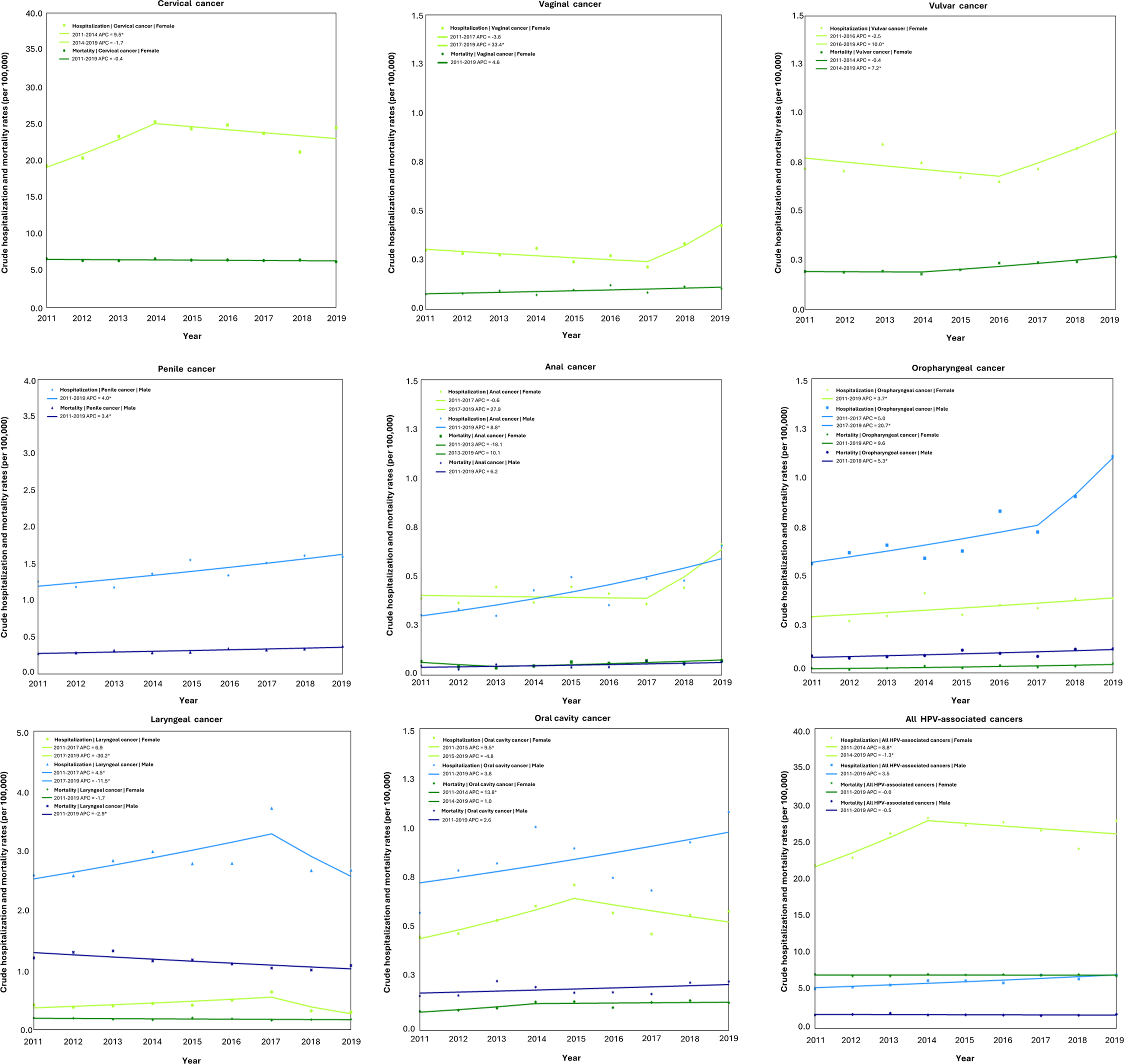

Supplement: S1 Fig — APC: Annual Percentage Change. Segments with significant APC values are marked with an asterisk; p ≤ 0.05. (TIF) [file pone.0335307.s008.tif]
