# Supplementary material for: Trends in the burden of HPV-associated cancers in Mexico: An analysis from 2011 to 2019
Source: PLoS One. 2025 Nov 13;20(11):e0335307. doi: 10.1371/journal.pone.0335307 (PMC12614612; doi:10.1371/journal.pone.0335307)
Supplement: S1 Appendix — (DOCX) [file pone.0335307.s009.docx]

**S1 Appendix. Data Sources**

This study used publicly available datasets from official Mexican government sources. All data are open access and freely available for download by any user without restriction. The authors had no special access privileges, and others can access the data in the same manner as the authors.

**1. Hospital Discharge Data (SAEH): Egresos Hospitalarios**

**Source:** Dirección General de Información en Salud (DGIS), Secretaría de Salud de México
**Dataset:** *Egresos Hospitalarios Sectorial*
**Period covered:** 2011–2019

**Download links:**

- [2011](http://www.dgis.salud.gob.mx/descargas/datosabiertos/egresos/sectorial_egresos_2011.zip?v=1.1)
- [2012](http://www.dgis.salud.gob.mx/descargas/datosabiertos/egresos/sectorial_egresos_2012.zip?v=1.1)
- [2013](http://www.dgis.salud.gob.mx/descargas/datosabiertos/egresos/sectorial_egresos_2013.zip?v=1.1)
- [2014](http://www.dgis.salud.gob.mx/descargas/datosabiertos/egresos/sectorial_egresos_2014.zip?v=1.1)
- [2015](http://www.dgis.salud.gob.mx/descargas/datosabiertos/egresos/sectorial_egresos_2015.zip?v=1.1)
- [2016](http://www.dgis.salud.gob.mx/descargas/datosabiertos/egresos/sectorial_egresos_2016.zip?v=1.1)
- [2017](http://www.dgis.salud.gob.mx/descargas/datosabiertos/egresos/sectorial_egresos_2017.zip)
- [2018](http://www.dgis.salud.gob.mx/descargas/datosabiertos/egresos/sectorial_egresos_2018.zip)
- [2019](http://www.dgis.salud.gob.mx/descargas/datosabiertos/egresos/sectorial_egresos_2019.zip)

**Variable descriptors:**

- [2004–2014](http://www.dgis.salud.gob.mx/descargas/datosabiertos/egresos/sectorial_Descriptores_Base_de_Datos_EGRESOS_2004_2014.xlsx?v=1.1)
- [2015–2016](http://www.dgis.salud.gob.mx/descargas/datosabiertos/egresos/sectorial_Descriptores_Base_de_Datos_EGRESOS_2015_2016.xlsx?v=1.1)
- [2017](http://www.dgis.salud.gob.mx/descargas/datosabiertos/egresos/sectorial_Descriptores_Base_de_Datos_EGRESOS_2017.xlsx?v=1.1)
- [2018–2024](http://www.dgis.salud.gob.mx/descargas/datosabiertos/egresos/sectorial_Descriptores_Base_de_Datos_EGRESOS_2018_2024.xlsx?v=2025.09.05)

**2. Mortality Data (INEGI and DGIS)**

**Source 1:** Instituto Nacional de Estadística y Geografía (INEGI)
**Dataset:** *Estadísticas de Defunciones Registradas (EDR)*
**Period covered:** 2011–2019

**Download links (INEGI):**

- [2011](https://www.inegi.org.mx/rnm/index.php/catalog/57/study-description)
- [2012](https://www.inegi.org.mx/app/descarga/ficha.html?tit=1969360&ag=0&f=csv)
- [2013](https://www.inegi.org.mx/app/descarga/ficha.html?tit=1969359&ag=0&f=csv)
- [2014](https://www.inegi.org.mx/app/descarga/ficha.html?tit=1969358&ag=0&f=csv)
- [2015](https://www.inegi.org.mx/app/descarga/ficha.html?tit=1969357&ag=0&f=csv)
- [2016](https://www.inegi.org.mx/app/descarga/ficha.html?tit=1969356&ag=0&f=csv)
- [2017](https://www.inegi.org.mx/app/descarga/ficha.html?tit=1969355&ag=0&f=csv)
- [2018](https://www.inegi.org.mx/app/descarga/ficha.html?tit=1969354&ag=0&f=csv)
- [2019](https://www.inegi.org.mx/app/descarga/ficha.html?tit=1969353&ag=0&f=csv)

**Source 2:** Dirección General de Información en Salud (DGIS), Secretaría de Salud de México
**Dataset:** *Defunciones – Registro de Defunciones*
**Period covered:** 2004–2019

**Download links (DGIS):**

- [2004–2011](http://www.dgis.salud.gob.mx/descargas/datosabiertos/defunciones/registro/DEFUN_2004-2011.zip?v=1.1.1)
- [2012–2013](http://www.dgis.salud.gob.mx/descargas/datosabiertos/defunciones/registro/DEFUN_2012-2013.zip?v=1.1.1)
- [2014](http://www.dgis.salud.gob.mx/descargas/datosabiertos/defunciones/registro/DEFUN_2014.zip?v=1.1.1)
- [2015](http://www.dgis.salud.gob.mx/descargas/datosabiertos/defunciones/registro/DEFUN_2015.zip?v=1.1.1)
- [2016](http://www.dgis.salud.gob.mx/descargas/datosabiertos/defunciones/registro/DEFUN_2016.zip?v=1.1.1)
- 2017
- [2018](http://www.dgis.salud.gob.mx/descargas/datosabiertos/defunciones/registro/DEFUN_2018.zip?v=1.1.1)
- [2019](http://www.dgis.salud.gob.mx/descargas/datosabiertos/defunciones/registro/DEFUN_2019.zip?v=1.1.1)

**Variable descriptors:**

- [2004–2011](http://www.dgis.salud.gob.mx/descargas/datosabiertos/defunciones/descriptores/DESCRIPTOR_CAMPOS_DEFUN_2004-2011.zip?v=1.1.1)
- [2012–2013](http://www.dgis.salud.gob.mx/descargas/datosabiertos/defunciones/descriptores/DESCRIPTOR_CAMPOS_DEFUN_2012-2013.zip?v=1.1.1)
- [2014](http://www.dgis.salud.gob.mx/descargas/datosabiertos/defunciones/descriptores/DESCRIPTOR_CAMPOS_DEFUN_2014.zip?v=1.1.1)
- [2015](http://www.dgis.salud.gob.mx/descargas/datosabiertos/defunciones/descriptores/DESCRIPTOR_CAMPOS_DEFUN_2015.zip?v=1.1.1)
- [2016](http://www.dgis.salud.gob.mx/descargas/datosabiertos/defunciones/descriptores/DESCRIPTOR_CAMPOS_DEFUN_2016.zip?v=1.1.1)
- [2017](http://www.dgis.salud.gob.mx/descargas/datosabiertos/defunciones/descriptores/DESCRIPTOR_CAMPOS_DEFUN_2017.zip?v=1.1.1)
- [2018](http://www.dgis.salud.gob.mx/descargas/datosabiertos/defunciones/descriptores/DESCRIPTOR_CAMPOS_DEFUN_2018.zip?v=1.1.1)
- [2019](http://www.dgis.salud.gob.mx/descargas/datosabiertos/defunciones/descriptores/DESCRIPTOR_CAMPOS_DEFUN_2019.zip?v=1.1.1)

Note: The INEGI and DGIS mortality datasets contain the same underlying vital statistics data, derived from the official national death registry. The difference lies in the organization and structure of the files provided by each institution.

**3. Population Estimates (CONAPO)**

**Source:** Consejo Nacional de Población (CONAPO)
**Dataset:** *Proyecciones de la Población de México y de las Entidades Federativas, 2020–2070*

**Access links:**

- [CONAPO official site](https://www.gob.mx/conapo)
- [Datos.gob.mx resource page](https://historico.datos.gob.mx/busca/dataset/proyecciones-de-la-poblacion-de-mexico-y-de-las-entidades-federativas-2020-2070/resource/14c2b735-2713-4a4a-b8f1-8c61dda8c579)
