# Supplementary material for: Trends in the burden of HPV-associated cancers in Mexico: An analysis from 2011 to 2019
Source: PLoS One. 2025 Nov 13;20(11):e0335307. doi: 10.1371/journal.pone.0335307 (PMC12614612; doi:10.1371/journal.pone.0335307)
Supplement: S2 Appendix — (DOCX) [file pone.0335307.s010.docx]

# S2 Appendix Python Script: Hospitalization ICD Extraction

#!/usr/bin/env python3

# -*- coding: utf-8 -*-

"""

Supplementary information – Hospitalization Data Extraction Script (EN)

Title: Trends in the burden of HPV-associated cancers in Mexico: an analysis from 2011 to 2019

Authors: Juan Carlos Orengo, Ana Luiza Bierrenbach, Carlos Eduardo Aranda Flores, Elsa Diaz Lopez, Julio Cesar Barbour Oliveira, Rodrigo Gonçalves Queijo, Cintia Irene Parellada*

Corresponding author: Cintia Irene Parellada (cintia.parellada@msd.com)

Description

-----------

Reads Mexican national hospitalization microdata (2011–2019), harmonizes sex and age,

maps ICD-10 codes to HPV-associated cancer groups, and outputs a tidy aggregated table by year, sex, age (years), and ICD group.

This study used publicly available datasets from official Mexican government sources. All data are open access and freely available for download by any user without restriction. The authors had no special access privileges, and others can access the data in the same manner as the authors.

Description

-----------

Reads the hospitalizations dataset for Mexico (2011–2019):

data_mx_hospitalizations_2011_2019.csv

and produces a tidy aggregated table by year, sex, age (years), and HPV-associated

ICD-10 group (counts of hospitalizations).

Expected input schema (detected):

- nm_SEXO : sex label in Spanish (e.g., "Femenino", "Masculino", "Intersexual", "Se ignora")

- AFECPRIN4 : principal diagnosis (ICD-10; typically 4 chars)

- EDAD : age in years (numeric). Missing/invalid ages are dropped.

- ano_proc : reference year (integer)

Outputs

-------

1) <basename>.xlsx or <basename>.csv (aggregated table)

2) <basename>.metadata.json (metadata with versions and counts)

3) S2_ICD_groups_reference.csv (catalog of ICD prefixes by group)

Usage

-----

python S1_Code_extract_hospitalization_ICD_Mexico_2011_2019.py \

--input "C:\\Users\\rodrigo.queijo\\Downloads\\Egresos Hospitalarios\\data_mx_hospitalizations_2011_2019.csv" \

--outdir "C:\\Users\\rodrigo.queijo\\Downloads\\Egresos Hospitalarios" \

--basename hospitalizations_mx_2011_2019_grouped \

--format xlsx \

--chunksize 500000

Requirements

------------

Python >= 3.9; pandas, numpy; openpyxl (for XLSX output)

License

-------

CC-BY 4.0

"""

from __future__ import annotations

import argparse

import json

import logging

import sys

from datetime import datetime

from pathlib import Path

from typing import Optional, Dict

import numpy as np

import pandas as pd

# ----------------------------------

# HPV-ASSOCIATED ICD-10 GROUPS (prefix-based)

# ----------------------------------

# Provide codes WITHOUT dots; matching uses startswith(), so subcategories map to the group.

ICD_GROUPS = {

"Anal Cancer": ["C21", "C21X", "C210", "C211", "C212", "C218"],

"Cervical Cancer": ["C53", "C53X", "C530", "C531", "C538", "C539"],

"Penile Cancer": ["C60", "C60X", "C600", "C601", "C602", "C608", "C609"],

"Vaginal Cancer": ["C52", "C52X"],

"Vulvar Cancer": ["C51", "C51X", "C510", "C511", "C512", "C518", "C519"],

"Laryngeal Cancer": ["C32", "C32X", "C320", "C321", "C322", "C323", "C328", "C329"],

"Oral Cavity Cancer": [

"C02", "C02X", "C020", "C021", "C022", "C023",

"C03", "C03X", "C030", "C031", "C039",

"C04", "C04X", "C040", "C041", "C048", "C049",

"C050", "C06", "C06X", "C060", "C061", "C062", "C068", "C069",

],

"Oropharyngeal Cancer": [

"C01", "C01X", "C024", "C051", "C052",

"C09", "C09X", "C090", "C091", "C098", "C099",

"C100", "C102", "C103", "C104", "C108", "C109",

],

}

SEX_ES_TO_EN = {

"HOMBRE": "Male", "MASCULINO": "Male",

"MUJER": "Female", "FEMENINO": "Female",

"INTERSEXUAL": "Intersex",

"SE IGNORA": "Unspecified", "IGNORADO": "Unspecified",

}

def parse_args():

p = argparse.ArgumentParser(description="Aggregate hospitalizations by HPV ICD groups.")

p.add_argument("--input", required=True)

p.add_argument("--outdir", default=".")

p.add_argument("--basename", default="hospitalizations_mx_2011_2019_grouped")

p.add_argument("--format", choices=["xlsx","csv"], default="xlsx")

p.add_argument("--chunksize", type=int, default=500_000)

return p.parse_args()

def setup_logging():

logging.basicConfig(level=logging.INFO, format="%(asctime)s %(levelname)s: %(message)s")

def _clean(s: str) -> str:

# Remove BOM and spaces, and convert to lowercase

return s.lstrip("\ufeff").strip().lower() if isinstance(s, str) else s

def resolve_columns(path: Path) -> Dict[str, str]:

"""Detect the real column names in the file (case-insensitive + BOM-safe)."""

hdr = pd.read_csv(path, dtype=str, nrows=0, engine="python", sep=None)

original_cols = list(hdr.columns)

normalized = {_clean(c): c for c in original_cols}

# Targets (standardized keys -> possible normalized aliases)

candidates = {

"nm_SEXO": ["nm_sexo", "sexo", "nmsexo"],

"AFECPRIN4": ["afecprin4", "afec_prin4", "afe_prin4", "afecprin", "cid", "icd", "icd10", "icd_10"],

"EDAD": ["edad", "edad1", "age", "edad_anios", "edad_anyos"],

"ano_proc": ["ano_proc", "ano", "ano_proceso", "year", "anio", "año", "ano_proc.", "ano_proces"],

}

resolved = {}

missing = []

for target, aliases in candidates.items():

found = None

for a in aliases:

if a in normalized:

found = normalized[a]; break

if found is None:

missing.append(target)

else:

resolved[target] = found

if missing:

# Debug help

logging.error("Header not matching. File columns: %s", original_cols)

raise ValueError(f"Columns not found (case-insensitive): {missing}")

logging.info("Resolved columns: %s", resolved)

return resolved

def normalize_icd(s: pd.Series) -> pd.Series:

return s.astype(str).str.upper().str.replace(".", "", regex=False).str.strip()

def classify_icd_group(code: Optional[str]) -> Optional[str]:

if not code or pd.isna(code): return None

for g, pre in ICD_GROUPS.items():

for p in pre:

if code.startswith(p):

return g

return None

def harmonize_sex_label(nm_sexo: pd.Series) -> pd.Series:

lab = nm_sexo.astype(str).str.strip().str.upper()

lab = lab.map(SEX_ES_TO_EN).fillna(lab)

lab = lab.replace({"FEMALE":"Female","MALE":"Male","INTERSEX":"Intersex","UNSPECIFIED":"Unspecified"})

return lab.where(lab.isin({"Male","Female","Intersex","Unspecified"}), "Unspecified")

def aggregate_streaming(path: Path, chunksize: int) -> pd.DataFrame:

# 1) Resolve true column names before using usecols

colmap = resolve_columns(path)

usecols = [colmap["nm_SEXO"], colmap["AFECPRIN4"], colmap["EDAD"], colmap["ano_proc"]]

chunks = []

total = 0

for i, chunk in enumerate(pd.read_csv(path, dtype=str, engine="python", sep=None, usecols=usecols, chunksize=chunksize)):

start = total; total += len(chunk)

logging.info("Processing lines %d–%d...", start, total)

# Rename to standardized internal names

chunk = chunk.rename(columns={

colmap["nm_SEXO"]: "nm_SEXO",

colmap["AFECPRIN4"]: "AFECPRIN4",

colmap["EDAD"]: "EDAD",

colmap["ano_proc"]: "ano_proc",

})

chunk["year"] = pd.to_numeric(chunk["ano_proc"], errors="coerce").astype("Int64")

chunk["age_years"] = pd.to_numeric(chunk["EDAD"], errors="coerce")

chunk = chunk[chunk["age_years"].notna()].copy()

chunk["sex"] = harmonize_sex_label(chunk["nm_SEXO"])

chunk["icd_clean"] = normalize_icd(chunk["AFECPRIN4"]).str[:5]

chunk["icd_group"] = chunk["icd_clean"].apply(classify_icd_group)

chunk = chunk[chunk["icd_group"].notna()].copy()

g = (chunk.groupby(["year","sex","age_years","icd_group"], dropna=False)

.size().reset_index(name="hospitalizations"))

chunks.append(g)

if not chunks:

logging.warning("No data loaded.")

return pd.DataFrame(columns=["year","sex","age_years","icd_group","hospitalizations"])

out = pd.concat(chunks, ignore_index=True)

out = (out.groupby(["year","sex","age_years","icd_group"], dropna=False)["hospitalizations"]

.sum().reset_index()

.sort_values(["year","icd_group","sex","age_years"], kind="mergesort"))

return out

def write_icd_catalog(out_dir: Path) -> Path:

rows=[]

for g, pre in ICD_GROUPS.items():

for p in sorted(set(pre)):

rows.append({"group": g, "icd_prefix": p, "note": "prefix match; dotless ICD"})

df = pd.DataFrame(rows)

out = out_dir / "S2_ICD_groups_reference.csv"

df.to_csv(out, index=False, encoding="utf-8-sig"); return out

def main():

args = parse_args(); setup_logging()

input_path = Path(args.input); out_dir = Path(args.outdir)

base = args.basename; fmt = args.format

if not input_path.exists():

logging.error("Input not found: %s", input_path); sys.exit(1)

logging.info("Reading merged hospitalizations: %s", input_path)

aggregated = aggregate_streaming(input_path, chunksize=args.chunksize)

out_dir.mkdir(parents=True, exist_ok=True)

out_path = out_dir / f"{base}.{fmt}"

if fmt == "xlsx":

try:

aggregated.to_excel(out_path, index=False)

except ModuleNotFoundError:

logging.error("openpyxl not found; use --format csv."); sys.exit(1)

else:

aggregated.to_csv(out_path, index=False, encoding="utf-8-sig")

meta = {

"created_utc": datetime.utcnow().isoformat(timespec="seconds")+"Z",

"script": str(Path(__file__).resolve()) if "__file__" in globals() else "<interactive>",

"input": str(input_path),

"rows_output": int(aggregated.shape[0]),

"columns_output": list(aggregated.columns),

"icd_groups": list(ICD_GROUPS.keys()),

"years_covered": "2011–2019",

"python_version": sys.version.split()[0],

"pandas_version": pd.__version__,

}

with open(out_dir / f"{base}.metadata.json","w",encoding="utf-8") as f:

json.dump(meta, f, ensure_ascii=False, indent=2)

icd_csv = write_icd_catalog(out_dir)

logging.info("[OK] Aggregated file: %s", out_path)

logging.info("[OK] ICD catalog: %s", icd_csv)

if __name__ == "__main__":

main()
