# Supplementary material for: Trends in the burden of HPV-associated cancers in Mexico: An analysis from 2011 to 2019
Source: PLoS One. 2025 Nov 13;20(11):e0335307. doi: 10.1371/journal.pone.0335307 (PMC12614612; doi:10.1371/journal.pone.0335307)
Supplement: S3 Appendix — (DOCX) [file pone.0335307.s011.docx]

# S3 Appendix Python Script: S3 Appendix - Mortality ICD Extraction

#!/usr/bin/env python3

# -*- coding: utf-8 -*-

"""

Supplementary information – Mortality Data Extraction Script (EN)

Title: Trends in the burden of HPV-associated cancers in Mexico: an analysis from 2011 to 2019

Authors: Juan Carlos Orengo, Ana Luiza Bierrenbach, Carlos Eduardo Aranda Flores, Elsa Diaz Lopez, Julio Cesar Barbour Oliveira, Rodrigo Gonçalves Queijo, Cintia Irene Parellada*

Corresponding author: Cintia Irene Parellada (cintia.parellada@msd.com)

Description

-----------

Reads Mexican national mortality microdata (2011–2019), harmonizes sex and age, maps ICD-10 codes to HPV-associated cancer groups, and outputs a tidy aggregatedtable by year, sex, age (years), and ICD group.

This study used publicly available datasets from official Mexican government sources. All data are open access and freely available for download by any user without restriction. The authors had no special access privileges, and others can access the data in the same manner as the authors.

Usage

-----

py S1_Code_extract_mortality_ICD_Mexico_2011_2019.py --input "C:\\Users\\rodrigo.queijo\\Downloads\\Defunciones\\data_mx_mortality_2011_2019.csv" --outdir "C:\\Users\\rodrigo.queijo\\Downloads\\Defunciones" --basename mortality_mx_2011_2019_grouped --format xlsx

Requirements

------------

- Python >= 3.9

- pandas, numpy

- openpyxl (only when writing XLSX)

Expected CSV Columns

--------------------

- SEXO: '1' (Male), '2' (Female), '9' (Unspecified)

- EDAD: encoded age (see function `edad_code_to_years`)

- CAUSA_DEF: ICD-10 basic cause of death

- YEAR_INGESTION: reference year used in this script

Assumptions

-----------

- EDAD codes representing days/weeks/months are treated as 0 years (i.e., <1 year).

- Rows with missing/invalid EDAD (age_years is NaN) are excluded from aggregation.

- YEAR_INGESTION is used as the time reference (the death year field).

Outputs

-------

1) <basename>.xlsx or <basename>.csv (aggregated table)

2) <basename>.metadata.json (metadata with versions and counts)

3) S2_ICD_groups_reference.csv (catalog of ICD prefixes by group)

License

-------

CC-BY 4.0

"""

from __future__ import annotations

import argparse

import json

import logging

import sys

from datetime import datetime

from pathlib import Path

from typing import Optional

import numpy as np

import pandas as pd

# ----------------------------------

# ICD GROUP DEFINITIONS (prefix-based)

# ----------------------------------

# NOTE: Codes must be provided WITHOUT dots. Matching is done by startswith(),

# which implies subcategories belong to the parent group (e.g., C320, C321 -> Laryngeal Cancer).

ICD_GROUPS = {

"Anal Cancer": ["C21", "C210", "C211", "C212", "C218"],

"Cervical Cancer": ["C53", "C530", "C531", "C538", "C539"],

"Penile Cancer": ["C60", "C600", "C601", "C602", "C608", "C609"],

"Vaginal Cancer": ["C52"],

"Vulvar Cancer": ["C51", "C510", "C511", "C512", "C518", "C519"],

"Laryngeal Cancer": ["C32", "C320", "C321", "C322", "C323", "C328", "C329"],

"Oral Cavity Cancer": [

"C02", "C020", "C021", "C022", "C023",

"C03", "C030", "C031", "C039",

"C04", "C040", "C041", "C048", "C049",

"C050", "C06", "C060", "C061", "C062", "C068", "C069",

],

"Oropharyngeal Cancer": [

"C01", "C024", "C051", "C052",

"C09", "C090", "C091", "C098", "C099",

"C100", "C102", "C103", "C104", "C108", "C109",

],

}

SEX_MAP = {"1": "Male", "2": "Female", "9": "Unspecified"}

# ----------------------------------

# ARGUMENT PARSING & LOGGING

# ----------------------------------

def parse_args() -> argparse.Namespace:

"""Define and parse command-line options."""

p = argparse.ArgumentParser(

description=(

"Aggregate Mexican mortality microdata (2011–2019) by sex, age (years), "

"and HPV-associated ICD-10 groups."

)

)

p.add_argument("--input", required=True, help="Path to the input CSV with microdata")

p.add_argument("--outdir", default=".", help="Output directory (default: current)")

p.add_argument("--basename", default="mortality_mx_2011_2019_grouped", help="Base name for output files")

p.add_argument("--format", choices=["xlsx", "csv"], default="xlsx", help="Output format (xlsx or csv)")

return p.parse_args()

def setup_logging() -> None:

"""Configure the root logger for consistent console messages."""

logging.basicConfig(level=logging.INFO, format="%(asctime)s %(levelname)s: %(message)s")

# ----------------------------------

# I/O HELPERS

# ----------------------------------

def robust_read_csv(path: Path) -> pd.DataFrame:

"""Read CSV with automatic encoding and separator detection.

We first try Latin-1 (common in government microdata) and fall back to UTF-8 with BOM.

The Python engine with `sep=None` lets pandas auto-detect the delimiter.

"""

read_kwargs = dict(dtype=str, engine="python", sep=None)

try:

return pd.read_csv(path, encoding="latin-1", **read_kwargs)

except UnicodeDecodeError:

return pd.read_csv(path, encoding="utf-8-sig", **read_kwargs)

# ----------------------------------

# DOMAIN TRANSFORMATIONS

# ----------------------------------

def edad_code_to_years(cve_str: Optional[str]) -> float:

"""Convert EDAD code to age in *years* (float).

Convention (based on the microdata dictionary):

- 4001–4120 => age in years (code = 4000 + age)

- 4998 => unknown/missing age -> NaN

- 1001–1098 (days), 2001–2098 (months), 3001–3098 (weeks)

=> return 0.0 (treated as <1 year) to include infants in year-based aggregation.

Any other value returns NaN.

"""

if cve_str is None or pd.isna(cve_str):

return np.nan

s = str(cve_str).strip()

if not s.isdigit():

return np.nan

cve = int(s)

if 4001 <= cve <= 4120:

return float(cve - 4000)

if cve == 4998:

return np.nan

if (1001 <= cve <= 1098) or (2001 <= cve <= 2098) or (3001 <= cve <= 3098):

# day / month / week → treat as <1 year

return 0.0

return np.nan

def normalize_icd(raw: pd.Series) -> pd.Series:

"""Normalize ICD-10 codes by uppercasing, removing dots, and trimming spaces."""

return raw.astype(str).str.upper().str.replace(".", "", regex=False).str.trim().fillna("")

# older pandas versions don't have Series.str.trim(); keep compatibility:

pd.Series.str.trim = getattr(pd.Series.str, "strip", pd.Series.str.strip)

def classify_icd_group(icd_code: Optional[str]) -> Optional[str]:

"""Return the HPV-associated group name if `icd_code` (dotless) starts with a known prefix.

This uses `startswith()` over the list of prefixes in `ICD_GROUPS`. Subcategories

(e.g., C320, C321) are captured by declaring parent prefixes (e.g., C32).

"""

if not icd_code or pd.isna(icd_code):

return None

for group, prefixes in ICD_GROUPS.items():

for p in prefixes:

if icd_code.startswith(p):

return group

return None

# ----------------------------------

# AGGREGATION

# ----------------------------------

def aggregate(df: pd.DataFrame) -> pd.DataFrame:

"""Aggregate deaths by (YEAR_INGESTION, sex, age_years, icd_group).

Notes

-----

- Keeps only records whose ICD maps to one of the HPV-associated groups.

- YEAR_INGESTION is used as the time reference.

- Rows with missing/invalid age (NaN) are excluded before aggregation.

"""

required = ["SEXO", "EDAD", "CAUSA_DEF"]

missing = [c for c in required if c not in df.columns]

if missing:

raise ValueError(f"Missing required columns: {missing}")

if "YEAR_INGESTION" not in df.columns:

raise ValueError("Column 'YEAR_INGESTION' not found in dataset.")

# Select only the necessary columns to reduce memory pressure on large files

d = df[required + ["YEAR_INGESTION"]].copy()

# YEAR_INGESTION may be string; coerce to integer (nullable) to handle bad rows gracefully

d["YEAR_INGESTION"] = pd.to_numeric(d["YEAR_INGESTION"], errors="coerce").astype("Int64")

# Harmonize sex codes; unknown values are mapped to "Unspecified"

d["sex"] = d["SEXO"].astype(str).str.strip().map(SEX_MAP).fillna("Unspecified")

# Convert EDAD codes to age in years (floats). Non-year codes become 0.0 (<1 year).

d["age_years"] = d["EDAD"].apply(edad_code_to_years)

# Exclude rows with missing/invalid age

d = d[d["age_years"].notna()]

# Normalize ICD (remove dots) then classify by prefix into HPV-related groups

d["icd_clean"] = normalize_icd(d["CAUSA_DEF"])

d["icd_group"] = d["icd_clean"].apply(classify_icd_group)

# Keep only mapped causes (drop non-HPV-related ICDs)

d = d[d["icd_group"].notna()].copy()

# Group and count

out = (

d.groupby(["YEAR_INGESTION", "sex", "age_years", "icd_group"], dropna=False)

.size()

.reset_index(name="deaths")

.sort_values(["YEAR_INGESTION", "icd_group", "sex", "age_years"], kind="mergesort")

)

out = out.rename(columns={"YEAR_INGESTION": "year_ingestion"})

return out

# ----------------------------------

# OUTPUT HELPERS

# ----------------------------------

def write_icd_catalog(out_dir: Path) -> Path:

"""Create CSV catalog of ICD prefixes per cancer group.

Adds a small note clarifying that matching is prefix-based over dotless ICD codes.

"""

rows = []

for group, prefixes in ICD_GROUPS.items():

for p in sorted(set(prefixes)):

rows.append({"group": group, "icd_prefix": p, "note": "prefix match over dotless ICD"})

df = pd.DataFrame(rows)

out_path = out_dir / "S2_ICD_groups_reference.csv"

df.to_csv(out_path, index=False, encoding="utf-8-sig")

return out_path

# ----------------------------------

# MAIN

# ----------------------------------

def main() -> None:

args = parse_args()

setup_logging()

input_file = Path(args.input)

output_dir = Path(args.outdir)

output_basename = args.basename

output_format = args.format

logging.info("Reading input: %s", input_file)

if not input_file.exists():

logging.error("File not found: %s", input_file)

sys.exit(1)

# Read input with robust encoding/separator detection

df = robust_read_csv(input_file)

# Normalize column names to uppercase (common in administrative data)

df.columns = [c.strip().upper() for c in df.columns]

# Inform about the first columns to help downstream users validate field names

preview_cols = ", ".join(df.columns[:15]) + (" ..." if len(df.columns) > 15 else "")

logging.info("Columns found (%d): %s", len(df.columns), preview_cols)

# Optional: log extra columns to make assumptions explicit

expected = {"SEXO", "EDAD", "CAUSA_DEF", "YEAR_INGESTION"}

extra = sorted(set(df.columns) - expected)

if extra:

logging.info("Extra columns ignored: %s", ", ".join(extra))

# Aggregate

aggregated = aggregate(df)

# Prepare outputs

output_dir.mkdir(parents=True, exist_ok=True)

output_path = output_dir / f"{output_basename}.{output_format}"

# Write aggregated table

if output_format == "xlsx":

try:

aggregated.to_excel(output_path, index=False)

except ModuleNotFoundError:

logging.error("openpyxl not found; install it or use --format csv.")

sys.exit(1)

else:

aggregated.to_csv(output_path, index=False, encoding="utf-8-sig")

# Write metadata alongside outputs (for reproducibility)

meta = {

"created_utc": datetime.utcnow().isoformat(timespec="seconds") + "Z",

"script": str(Path(__file__).resolve()) if "__file__" in globals() else "<interactive>",

"input": str(input_file),

"rows_output": int(aggregated.shape[0]),

"columns_output": list(aggregated.columns),

"icd_groups": list(ICD_GROUPS.keys()),

"years_covered": "2011–2019",

"python_version": sys.version.split()[0],

"pandas_version": pd.__version__,

}

with open(output_dir / f"{output_basename}.metadata.json", "w", encoding="utf-8") as f:

json.dump(meta, f, ensure_ascii=False, indent=2)

# Write ICD catalog

icd_csv = write_icd_catalog(output_dir)

logging.info("[OK] Aggregated table saved: %s", output_path)

logging.info("[OK] ICD catalog saved: %s", icd_csv)

# Print a deterministic sample of rows to help data reviewers

pd.options.display.width = 0 # avoid line wrapping/truncation in terminals

sample_n = min(20, len(aggregated))

if sample_n > 0:

print(aggregated.sample(sample_n, random_state=42).to_string(index=False))

if __name__ == "__main__":

main()
